# Lysosome-dependent cell death and deregulated autophagy induced by amine-modified polystyrene nanoparticles

**DOI:** 10.1098/rsob.170271

**Published:** 2018-04-11

**Authors:** Fengjuan Wang, Anna Salvati, Patricia Boya

**Affiliations:** 1 Center for BioNano Interactions, School of Chemistry and Chemical Biology, University College Dublin, Belfield, Dublin 4, Ireland; 2 CNRS-University of Strasbourg, Biotechnology and cell signaling, France/Laboratory of excellence Medalis, Illkirch, France; 3 Groningen Research Institute of Pharmacy, Groningen University, Antonius Deusinglaan 1, Groningen 9713AV, The Netherlands; 4 Autophagy Lab, Department of Cellular and Molecular Biology, Centro de Investigaciones Biológicas, CSIC, Ramiro de Maeztu 9, 28040 Madrid, Spain

**Keywords:** cationic nanoparticles, lysosomal membrane permeabilization, autophagy, reactive oxygen species, nanotoxicity

## Abstract

Nanoparticles (NPs) typically accumulate in lysosomes. However, their impact on lysosomal function, as well as autophagy, a lysosomal degradative pathway, is still not well known. We have previously reported in the 1321N1 cell line that amine-modified polystyrene (NH_2_-PS) NPs induce apoptosis through damage initiated in the lysosomes leading ultimately to release of lysosomal content in the cytosol, followed by apoptosis. Here, by using a combination of biochemical and cell biological approaches, we have characterized in a mouse embryonic fibroblast cell line that the lysosomal alterations induced by NH_2_-PS NPs is progressive, initiating from mild lysosomal membrane permeabilization (LMP), to expansion of lysosomal volume and intensive LMP before the summit of cell death. Though the cells initially seem to induce autophagy as a surviving mechanism, the damage of NH_2_-PS NPs to lysosomes probably results in lysosomal dysfunctions, leading to blockage of autophagic flux at the level of lysosomes and the eventual cell death.

## 1. Introduction

Damage to lysosomes has recently been proposed as an emerging mechanism of nanotoxicity [1,2], as most endocytosed nanoparticles (NPs) accumulate within the lysosomal compartments without evident exit [3–5]. Evaluating lysosomal function after NP accumulation in the lysosomes is important to analyse the toxicological consequences of NPs [2,6]. The so-called ‘protein corona', namely layers of proteins and other biomolecules (adsorbed from the cell medium) on the surface of NPs [7,8], also needs to be considered when examining nanotoxicity [9,10]. The composition of the corona is highly dependent on the properties of NPs and media. When exposed to NPs, the cells ‘see' the NP/corona complexes, but not the pristine NPs *per se* [11,12]. Using amine-modified polystyrene (NH_2_-PS) NPs as an example, we have previously shown that the NP/corona complexes enter cells together and home in lysosomes [9,13]. Once inside lysosomes, the corona gets degraded by lysosomal enzymes. The degradation of the original corona layer is accompanied by strong lysosomal alterations [9,14,15]. Although several reports have proposed the so-called ‘proton sponge' effect as the mechanism of lysosomal damage by nanomaterials [16,17], similar effects have been reported also for materials not capable of buffering the lysosomal pH [9,18]. Other mechanisms have also been proposed, involving for instance damage to chloride channels [19].

Lysosomal alterations are tightly related with lysosomal dysfunction and have been shown to be crucial in a plethora of cell death scenarios and pathological contexts [20,21]. Lysosome-dependent cell death proceeds upon lysosomal membrane permeabilization (LMP), resulting in the release of lysosomal contents, including proteolytic enzymes of the cathepsin family, to the cytoplasm [20,22]. Moreover, lysosomal alterations can be associated with deregulation of autophagy in cell death and diseases [20,23,24]. Autophagy is a self-digestive process dependent on lysosomal degradation, and it is classified as macroautophagy, chaperone-mediated autophagy and microautophagy. In macroautophagy (hereafter referred to as autophagy), a double membrane structure is generated to engulf some cytosolic components (such as damaged proteins and organelles) to form autophagosomes. The resulting autophagosomes further fuse with lysosomes to form autolysosomes, in which lysosomal proteases could degrade the engulfed components inside autophagolysosomes [25,26]. Therefore, when lysosomes suffer dysfunction, fusion between autophagosomes and lysosomes and/or degradation of autophagosomes is compromised, affecting autophagy.

The widely used method to analyse autophagy is the detection of the lipidated form of the microtubule-associated protein 1 light chain 3, or LC3-II, as it is recruited to the membrane of autophagosomes. The amount of LC3-II is relative to the amount of autophagosomes [27]. However, both induction and blockage of autophagy could result in the increase of LC3-II level [27,28]. The more precise autophagy analysis is therefore to measure autophagic flux (or the rate of autophagy), in which the turnover of LC3-II is analysed in the presence and absence of lysosomal inhibitors, such as chloroquine, bafilomycin A and protease inhibitors [27,29].

A number of NPs have been reported to either activate or block autophagy, as is summarized in the review of Stern *et al*. [2,30]. The autophagy modulating property of NPs can, on the one hand, be employed for drug targeting purposes [1,31], while on the other hand, it has been suggested as a toxicity mechanism of NPs [2]. Nevertheless, very often the possibility of autophagy blockage induced by NPs was overlooked, as most detection of autophagy was done by measurement of LC3-II level alone, as is also mentioned in the review of Stern *et al*. [2]. Moreover, in order to be able to determine the flux, this kind of assay should be done over time, rather than just at a single time point with different NP doses [32].

Here, using mouse embryonic fibroblast (MEF), by combining fluorescence imaging, flow cytometry and cell fractionation assays, we demonstrate that NH_2_-PS NPs cause progressive lysosomal alterations, from earlier mild LMP to later lysosomal expansion and massive LMP. We also decipher what are the cell death ‘signals’ coming from the ‘leaking' lysosomes that exacerbate cell death. Furthermore, we describe how autophagy was affected during the dynamic change of lysosomal status after NP treatment.

## 2. Results

### 2.1. Nanoparticles induce lysosomal alterations and cell death

#### 2.1.1. Nanoparticles accumulate in lysosomes and cause lysosomal expansion and cell death

**Figure 1.**
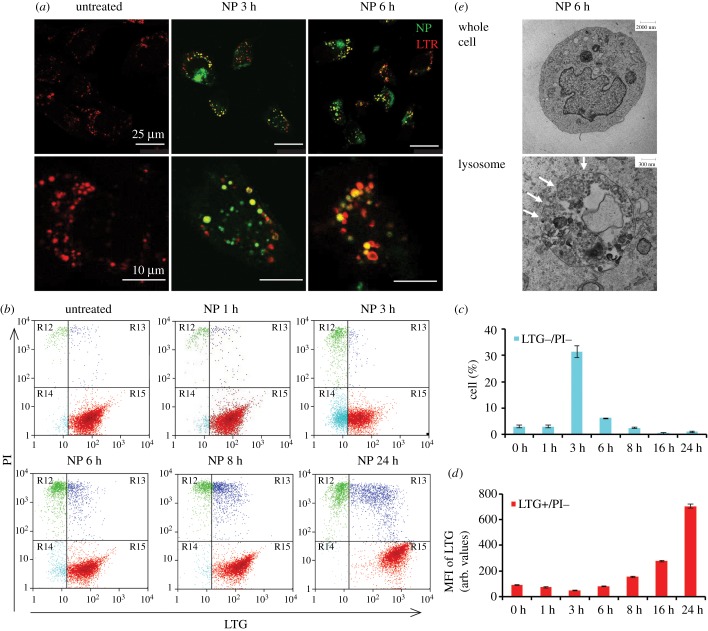
Confocal imaging and flow cytometric analysis show NH_2_-PS NPs induce lysosomal damage. (*a*) Confocal images of LTR staining. MEF cells were treated with NH_2_-PS NPs (green) for 0, 3 and 6 h and stained with LTR (red). The images indicate accumulation of NPs into lysosomes at 3 h and lysosomal swelling at 6 h. Zoomed images of cells are shown in the bottom panel. The quantification of lysosomal size is shown in electronic supplementary material, figure S1. (*b*) Flow cytometric analysis of LTG/PI double staining. MEF cells were treated with NH_2_-PS NPs for indicated time points and stained with LTG and PI. The dot plots of PI versus LTG are illustrated. (*c*) The percentage (%) of LTG−/PI− cells in (*b*) at indicated time points. (*d*) The MFI of LTG of LTG+/PI− cells in (*b*) at indicated time points. Results are the mean of three experiments, each with two replicates, and the error bars are the standard deviations. (*e*) TEM images of 1321N1 cells treated with NPs for 6 h. A whole cell is shown in the top panel and a lysosome from this cell is shown in the bottom panel. White arrows indicate permeabilized lysosomal membrane.

We and others have shown that most NH_2_-PS NPs enter cells by endocytosis and are delivered to lysosomes in several human cell lines [5,9,14,33]. We have examined the cellular localization of NH_2_-PS NPs in MEF cells by confocal fluorescence imaging. MEF cells were stained with the lysosomotropic dye-LysoTracker Red (LTR) that selectively accumulates in the cellular acidic compartments (mainly lysosomes). The confocal images in figure 1*a* illustrate that some NH_2_-PS NPs (which fluoresce in blue channel but are coloured in green here for better visualization) can be found to colocalize with LTR (in red) as soon as 3 h exposure, confirming that NH_2_-PS NPs accumulate to lysosomes in MEF cells, in agreement with what has been observed in other cell types. Strikingly, after 6 h exposure to NH_2_-PS NPs, the LTR positive vesicles significantly expand (electronic supplementary material, figure S1), indicative of lysosomal swelling, similar to the observations in other cell types [9,14].

To further investigate the effect of NH_2_-PS NPs on lysosomal function and cell viability, cells were co-stained with the lysosomotropic dye-LysoTracker Green (LTG) and viability dye-propidium iodide (PI), followed by flow cytometry measurement. Untreated cells show positive lysosomal staining and no rupture of the plasma membrane (LTG+/PI−) (figure 1*b*). After 3 h exposure to NH_2_-PS NPs, we have first observed a reproducible increase of cells with mild or partial LMP but still intact plasma membrane (LTG−/PI−, coloured in cyan) (figure 1*b*,*c*), similar to the onset of lysosomal-dependent cell death reported elsewhere [20,23].

We have included the positive control *t*-BuOOH, a classical LMP inducer that causes lysosomal damage via oxidative stress [20,21]. The percentage of LTG−/PI− cell population increases with longer exposure time and higher concentration of *t*-BuOOH (electronic supplementary material, figure S2, coloured also in cyan), supporting that the LTG−/PI− cell population after 3 h exposure to NPs shows LMP. Cells eventually become LTG−/PI+ (coloured in green; electronic supplementary material, figure S2) after *t*-BuOOH treatment, which corresponds to total rupture of lysosomes and late stage cell death.

Intriguingly, this LTG−/PI− population (in cyan) cannot be observed at longer exposure time to NH_2_-PS NPs (figure 1*b*, NP 6 h, 8 h and 24 h; figure 1*c*; electronic supplementary material, figure S3A). Instead, the LTG intensity of the LTG+/PI− (in red) population increases over time, as is quantified in figure 1*d* and electronic supplementary material, figure S3B. This result is coherent with the lysosomal swelling phenomenon, observed with confocal fluorescence imaging in figure 1*a* and previously reported data obtained with the same particles in 1321N1 cells [14].

Similar data were obtained with a different dye combination, namely lysosomotropic dye LTR and viability dye TO-PRO-3, to exclude the artefacts potentially caused by interference between fluorescence dyes. The results (electronic supplementary material, figure S4) also show a population with LTR−/TO-PRO-3- (highlighted in the cyan box) upon 3 h exposure to NH_2_-PS NPs, with an increase of LTR intensity of LTR+/TO-PRO-3- at the later exposure time points, confirming the above results obtained with LTG/PI staining.

We further assessed the destabilization of lysosomes after NP treatment by ultrastructure transmission electron microscopy (TEM) analysis. Polystyrene NPs have an electron density very similar to cells and could be very difficult to detect once internalized. However, careful observations and comparison with control cells allow us to define structures that are likely to be NPs inside endolysosomes (ELs). Interestingly, in some cases abnormal morphology of ELs was also observed after exposure to NH_2_-PS NPs. In addition, some NP-loaded ELs displayed clear interruptions of their membrane, indicative of LMP (figure 1*e*; electronic supplementary material, figures S5 and S6, white arrows).

Together these data show dramatic and dynamic alterations in lysosomal morphology after NP treatment, from mild LMP to lysosomal swelling and destabilization.

#### 2.1.2. Nanoparticles induce lysosomal membrane permeabilization and cathepsin release

**Figure 2.**
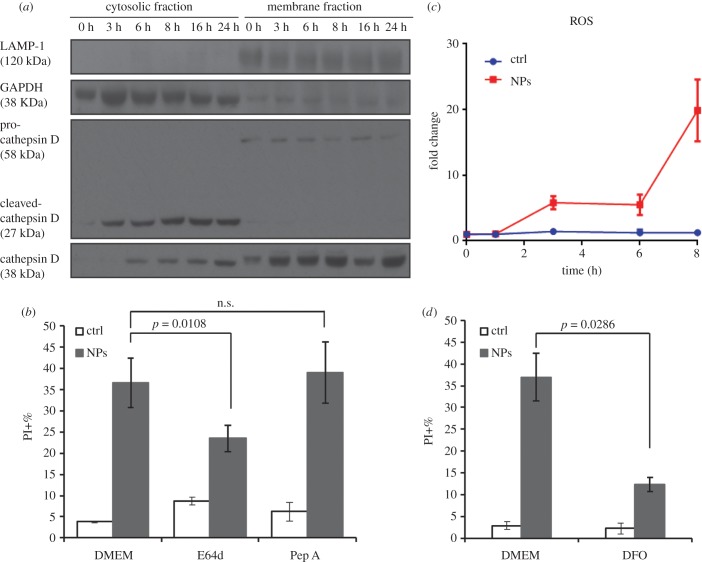
Cytosolic release of lysosomal proteases upon exposure of NH_2_-PS NPs and lysosomal cell death. (*a*) Western blot of cytosolic release of lysosomal proteases. MEF cells were treated with NPs for indicated time points, followed by cell fractionation to obtain cytosolic and membrane fractions. Both fractions were subjected to western blot to detect indicated proteins. LAMP1 is a lysosomal marker and GAPDH is a cytosolic marker, the results of which show no cross-contamination of cytosolic and lysosomal fractions. Cathepsin D and B can be detected in the cytosolic fraction after 3 h and 6 h exposure to NPs, respectively. (*b*) Cell death induced by NH_2_-PS NPs with or without lysosomal protease inhibitors. The percentages of PI positive cells were measured by flow cytometry. (*c*) ROS generation upon exposure to NH_2_-PS NPs. MEF cells were treated with NPs as described above at indicated time points, followed by staining of 2.5 µM CM-H_2_DCFDA and flow cytometric measurement. The MFI of cellular CM-H_2_DCFDA fluorescence is normalized by that of untreated cells at time 0. The fold changes are plotted here. (*d*) Cell death caused by NH_2_-PS NPs in the presence or absence of lysosomal iron chelator DFO. Results are the mean values of three experiments, each with two replicates, and the error bars are the standard deviations. One-way ANOVA was used to analyse the statistical significance.

The characteristic sign of LMP is the release of lysosomal content, such as cathepsins, into the cytosol [20]. Lysosomal proteases cathepsin B and D normally reside within the lysosomal lumen, and upon LMP they can be released to cytosol, leading to cell death [20,34]. To verify the mild LMP after 3 h exposure to NH_2_-PS NPs, we performed cell fractionation, followed by western blot, to detect cathepsin B and D in cytosolic fractions of cells after exposure to NH_2_-PS NPs. Cathepsin D has an unprocessed form (58 kDa) and a processed form (27 kDa). The smaller processed form was clearly observed in the cytosolic fraction after 3 h exposure to NPs (figure 2*a*), confirming that the LTG−/PI− population observed above (figure 1*b*,*c*) also after 3 h exposure time to these NPs is indeed a cell population with LMP. After 6 h exposure to NH_2_-PS NPs, cathepsin B (38 kDa) can also be detected in cytosolic fractions (figure 2*a*). The cytosolic levels of both cathepsins increase with exposure time to NH_2_-PS NPs, consistent with the progressive lysosomal damage observed with LTG/PI staining in figure 1*b* and electronic supplementary material, figure S3.

#### 2.1.3. NH_2_-PS nanoparticles induce lysosomal-dependent cell death

Release of cathepsins from lysosomes to cytosol can lead to various apoptosis signalling [20,35–37]. We have observed that these NPs induce early apoptosis at around 8 h in MEF cells as AnnexinV+/PI− cells can be detected (electronic supplementary material, figure S7). As expected, at later time points, apoptosis proceeds to necrosis, where most of the cells are AnnexinV+/PI+. This is in line with what we have previously observed in 1321N1 cells [14,15]. To continue, we examined whether the cell death induced by NH_2_-PS NPs was dependent on cathepsins by using cathepsin inhibitors. Since we have observed the release of cathepsin B and D into cytosol upon NP treatment, we used E64d and pepstatin A (Pep A) to inhibit their activities, respectively. Cells were exposed to NH_2_-PS NPs with or without these inhibitors, and the percentage of cell death was measured by flow cytometry analysis of PI staining (figure 2*b*). The results show that E64d decreases the cell death by 20% as compared with cells treated by NH_2_-PS NPs alone, which may suggest a specific role for cathepsin B in the cell death induced by these NPs. Inhibition of cathepsin D by Pep A however does not seem to affect the extent of cell death induced by these NPs, suggesting that either this drug is less effective [38] or that cathepsin D plays a minor role in propagating the damage and leading to cell death.

ROS is a well-known inducer of LMP [20,36]. Interestingly, many NPs are known as ROS generators due to their reactive particle surface [16,39]. However, it is essential to distinguish this primary ROS generated at the surface of NPs from secondary ROS originated in cells as a consequence of cell damage of a different nature (rather than a direct reaction on the NP surface). In this context, NH_2_-PS NPs do not have the capacity to produce ROS directly on their surface [40]. Thus, in this case, eventual ROS in cells is due to the damage they induce on cell structures, such as lysosomes and mitochondria. We have previously studied in detail whether ROS are generated before or after the observed lysosomal alterations induced by these NPs. With a time-resolved study, we could determine that ROS is generated downstream as a consequence of the release of the lysosomal content in the cytosol in 1321N1 cells [14]. Here we examined if NH_2_-PS NPs induced ROS in MEF cells as well. In line with our previous observations in 1321N1 cells, we have observed that in MEF cells NH_2_-PS NPs induce a fivefold increase of ROS measured by CM-H_2_DCFDA, after 3 h incubation of NPs (figure 2*c*), thus in the same time range in which NPs are trafficked to lysosomes. A dramatic 20-fold increase of ROS can be observed after 8 h exposure to NPs, indicating a substantial mitochondrial damage (figure 2*c*; electronic supplementary material, figure S8). Indeed, TEM images show that after 8 h NP treatment, mitochondria lost their cristae, indicative of damage to mitochondria (electronic supplementary material, figure S9). This massive amount of ROS probably further amplifies the damage induced by these NPs, leading ultimately to cell death [20,36].

Oxidative damage to lysosomes can be catalysed by intralysosomal low mass iron, a majority part of cellular redox-active iron, which sensitizes lysosomal membrane and leads to eventual lysosomal rupture and cell death. Iron chelators, such as desferrioxamine (DFO), have been shown to protect cells from oxidative stress [41]. DFO enters cells via endocytosis, and accumulates to lysosomes, where it acts as a lysosomal ROS inhibitor by chelating lysosomal redox-active iron. Pre-incubation with DFO in MEF cells significantly reduces the cell death induced by NH_2_-PS NPs (figure 2*d*), confirming that the generation of ROS following exposure to NH_2_-PS NPs is key in the following propagation of cell damage leading to cell death.

### 2.2 Nanoparticles affect autophagic pathway

#### 2.2.1. Nanoparticles alter autophagic flux

**Figure 3.**
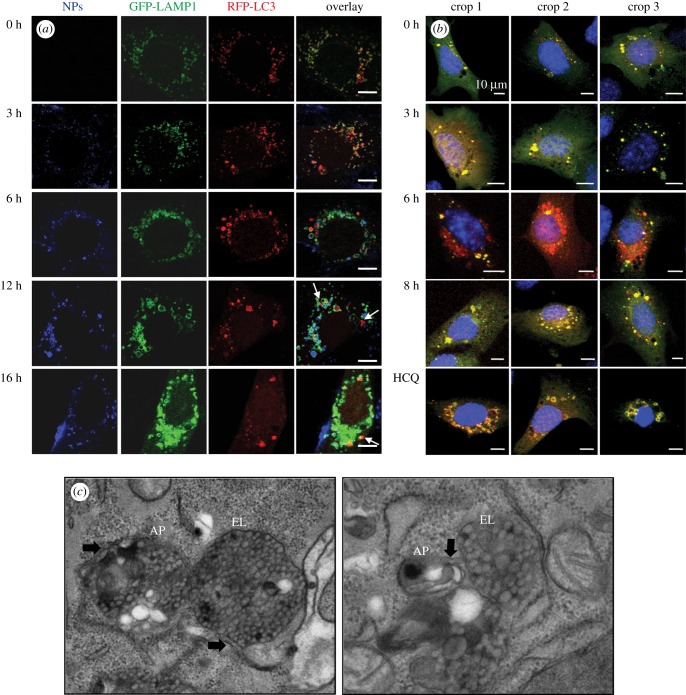
NH_2_-PS NPs induce accumulation of autophagosomes. (*a*) Confocal images of autophagosomes and lysosomes after NP treatment. MEF cells were co-transfected with LC3-RFP (red) and LAMP-1 (green), and exposed to NH_2_-PS (blue) for indicated time points. White arrows indicate the autophagosomes that do not colocalize with lysosomes. The scale bar is 10 µm. (*b*) Confocal images of MEF cells transfected with tf-LC3 plasmid followed by exposure to NH_2_-PS NPs for indicated times or HCQ. (*c*) TEM images of autophagic vesicles. AP, autophagosome; EL, endolysosome. Black arrows indicate the double membrane structures of AP. The magnification levels of the images are 50 000× and 80 000×, respectively.

Lysosomal alterations could directly affect autophagy [23]. We therefore examined the generation of autophagosomes and their fusion with lysosomes, by co-transfecting MEF cells with LC3-RFP (red) plasmid to label autophagosomes and lysosomal associated membrane protein 1 (LAMP1)-GFP (green) plasmid to label lysosomes, after exposure to NH_2_-PS NPs (visualized as blue). The confocal fluorescence images show that most NH_2_-PS NPs colocalize with LAMP1-GFP and the enlargement of lysosomal compartments can be observed after 6 h exposure to NH_2_-PS NPs (figure 3*a*; electronic supplementary material, figure S10), consistent with the results obtained by LTR staining in figure 1*a*. The LC3-RFP vesicles colocalize with LAMP1-GFP in the earlier hours (0, 3 and 6 h), suggesting that autophagosomes, at this stage, are capable of fusing with lysosomes. However, at 12 h and 16 h, there seems to be more LC3-RFP-positive autophagosomes (with much bigger size) that do not colocalize with LAMP1-GFP positive lysosomes (white arrows).

To further examine autophagosomes and their fusion with lysosomes after treatment of NH_2_-PS NPs, MEF cells were transfected with RFP-GFP tandem fluorescent-tagged LC3 (tf-LC3) plasmid that allows for autophagy flux assessment [42]. After transfection, autophagosomes show yellow fluorescence, because LC3 is conjugated with both RFP and GFP; when autophagosomes fuse with lysosomes to form autolysosomes, the GFP gets quenched in the acidic lysosomes, therefore autolysosomes show red fluorescence [42]. In the untreated cells, the numbers of yellow (autophagosomes) and red (autolysosomes) punctate structures are both low (figure 3*b*), which corresponds to the basal level of autophagy. After 6 h NP treatment, more red autolysosomes can be observed, indicative of functional autophagic flux at this stage, which leads to the quenching of the GFP fluorescence of tf-LC3 in autolysosomes. However, after 8 h exposure to NH_2_-PS NPs, mainly yellow autophagosomes can be detected, similar to the accumulation of yellow autophagosomes after treatment of hydrochloroquine (HCQ) that is known to elevate lysosomal pH and block autophagic flux [43–45]. Combining the results of LC3-RFP/LAMP1-GFP and tf-LC3, we suspect that a blockage of autophagy is likely after 8 h exposure to NH_2_-PS NPs.

We have also performed TEM to examine the morphology of autophagosomes and ELs. We have observed a massive accumulation of double and multiple membrane structures, namely autophagosomes (APs) and multivesicular bodies (MVBs) [46], in the cells treated by NH_2_-PS NPs for 8 h (figure 3*c*; electronic supplementary material, figure S11A). Moreover, we have observed that vesicles containing NH_2_-PS NPs (probably endosomes or lysosomes) are surrounded by double membrane structures (black arrows in figure 3*c*, left panel; electronic supplementary material, figure S11A), suggesting that they could be amphisomes (formed by fusion between autophagosomes and endosomes) or autolysosomes.

**Figure 4.**
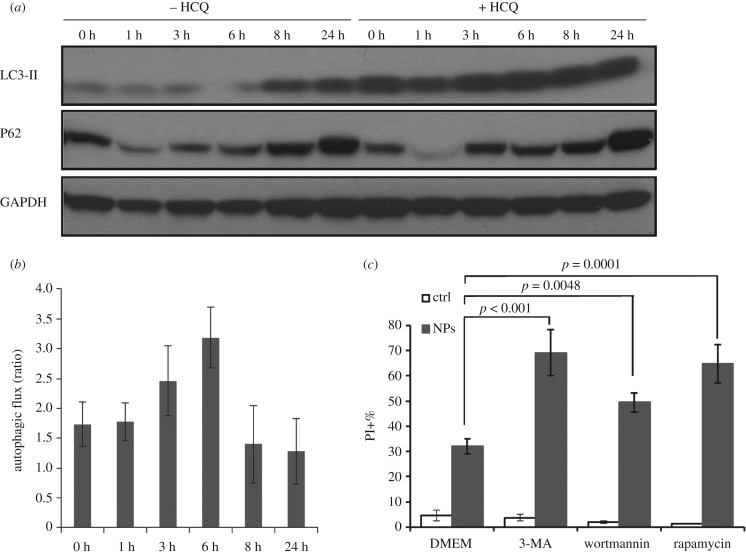
Treatment of NH_2_-PS NPs blocks autophagic flux. (*a*) Western blot of autophagy markers in MEF cells treated with NPs with or without HCQ. The LC3-II levels were plotted in electronic supplementary material, figure S11B. Higher exposure time for the revelation of LC3 western blot is shown in electronic supplementary material, figure S11C. (*b*) Analysis of autophagic flux. The autophagic flux was calculated as ratios via dividing the levels of LC3-II after NP treatment in the presence of HCQ by that in the absence of HCQ. Results are mean values from two independent experiments, and the error bars are standard deviations. (*c*) Cell death induced by NH_2_-PS NPs in the presence of pharmacological modulators of autophagy. MEF cells were treated by NH_2_-PS NPs for 24 h with or without autophagy inhibitors (3-MA or wortmannin) or an autophagy inducer (rapamycin). The percentages of PI positive cells were plotted. Results of the effects of the inhibitors on 8 h treatment with NPs are shown in electronic supplementary material, figure S12. Results are mean values from three independent experiments with two replicates each, and the error bars are standard deviations. One-way ANOVA was used to analyse the statistical significance.

Finally, we have evaluated the autophagic flux through western blot detection of LC3-II in the presence or absence of lysosomal inhibitors and the levels of the autophagy substrate p62. We have observed that NP treatment can increase the levels of LC3-II after 8 h exposure to NH_2_-PS NPs (figure 4*a*; electronic supplementary material, figure S11B), confirming the accumulation of autophagosome after 8 h NP treatment. The accumulation of LC3-II can be either induction or blockage of autophagic flux [29]. To assess the autophagic flux, we have compared the difference of LC3-II levels after NP treatment with or without lysosomal inhibitor HCQ over time. The resulting plot indicates that autophagic flux is slightly induced after 3–6 h treatment of NH_2_-PS NPs; however, after 8 and 24 h exposure to NH_2_-PS NPs, there is a decrease of autophagic flux (figure 4*b*). This correlates with the western blot detection of p62 (figure 4*a*), a substrate which is degraded by autophagy [47]. We have first observed a decrease of p62 in the first 6 h of NP treatment (indicative of induced autophagic flux), followed by an increase of its amount after 8 and 24 h (reflecting the blockage of autophagic flux). Together with the fluorescence images shown above (figure 3*a*,*b*), our data strongly indicate that in the first 6 h exposure to NPs, autophagy is functional, while after 8 h NPs induce blockage of autophagic flux, probably due to the fact that lysosomes are severely damaged at this time.

#### 2.2.2. Nanoparticles interact with PI3 K/AKT/mTOR signalling pathway

The phosphatidylinositol-3-kinase (PI3 K)/AKT/mTOR (mammalian target of rapamycin) signalling pathway is known to regulate autophagy in response to nutritional status and other stimuli [48]. PI3 K activated by pro-surviving signals could in turn activate and phosphorylate AKT. Activated AKT signals to mTOR, which activates and phosphorylates its downstream effector p70S6 kinase (S6 K). This pathway negatively regulates autophagy [49]. Therefore, the amount of phosphorylated ATK and S6 K (pATK and pS6 K) negatively correlate with autophagy activation. After exposure to NH_2_-PS NPs, we have observed a decrease of pAKT at 8 h and pS6 K at 24 h (electronic supplementary material, figure S11C), indicative of inhibition of mTOR and activation of autophagy. This is consistent with the induction of autophagy observed above. Similar activation of autophagy through PI3 K/AKT/mTOR signalling pathway after exposure to NH_2_-PS NPs has been observed in RAW 264.7 and BEAS-2B cells [50]. In conclusion, NH_2_-PS NPs regulate mTOR-dependent autophagy.

#### 2.2.3. Inhibition of autophagy sensitizes cells to nanoparticle treatment

The role of autophagy can be different in different cell death scenarios. In many cases, autophagy serves as a survival mechanism to cope with the cellular stress; however, in other cases, cells also die with autophagic features [51,52]. Here, we examine the role of cell death with pharmacological inhibitors of autophagy, including both 3-methyladenine (3-MA) and wortmannin. When MEF cells were treated with NH_2_-PS NPs in the presence of 3-MA or wortmannin, we observed an elevated level of cell death (figure 4*c*; electronic supplementary material, figure S12), indicating that autophagy is a pro-survival mechanism in the cell death induced by NH_2_-PS NPs. This result also supports the results of inhibition of mTOR after 8 h (§2.2.2) that autophagy is indeed activated in order to deal with the damage induced by NPs. Interestingly, autophagy induction with rapamycin augmented cell death (figure 4*c*; electronic supplementary material, figure S12), suggesting that increasing autophagosome formation under conditions of lysosomal dysfunction is detrimental for cell survival. In all, lysosomes are no longer functional after NP treatment and autophagy fails to save the cells from the NP-induced stress, leading to accumulation of autophagosomes and eventual cell death [23,42].

#### 2.2.4. Impact of nanoparticles on autophagy in 1321N1 cells

We have previously demonstrated that in 1321N1 cells, NH_2_-PS NPs induce accumulation of autophagosomes by both western blot and confocal fluorescent imaging of LC3-II [14]. Here, we have complemented this study by examining autophagosomes via TEM, assessing autophagic flux and monitoring PI3 K/ATK/mTOR signalling pathway in parallel with the above studies in MEF cells. First, TEM (electronic supplementary material, figure S13A) results show that double and multiple membrane structures can be found after NP treatment in 1321N1 cells, confirming the accumulation of autophagosomes. Then we have analysed the autophagic flux in 1321N1 cells after exposure to NH_2_-PS NPs. The analysis of LC3-II levels upon NP exposure with or without HCQ suggests that the autophagic flux is decreased after NH_2_-PS NPs treatment even after 1 h exposure to NH_2_-PS NPs (electronic supplementary material, figure S13*b*,*c*), indicating that the accumulation of LC3-II is due to blockage of autophagy after NP treatment. We have also tested E64d as a lysosomal inhibitor, and we have observed no increase of LC3-II in the presence of E64d upon exposure to NH_2_-PS NPs compared with that of NP treatment alone (electronic supplementary material, figure S13D), supporting that autophagic flux was indeed blocked upon NP treatment. p62 level was also assessed, and we have observed only a small decrease of p62 after 1 h treatment, and the amount increases after 3 h NP treatment (figure 13B), confirming the blockage of autophagy after exposure to NH_2_-PS NPs at this exposure time. The decrease of pAKT and pS6 K has been observed at 6 h exposure to NH_2_-PS NPs. All these results suggest that in both MEF and 1321N1 cells, autophagy could be activated upon NP exposure; however, the final consequence is the overall blockage of autophagic flux, due to the severe damage to lysosomes.

## 3. Discussion

### 3.1. The time profile of lysosome and autophagy alternations induced by nanoparticles

**Figure 5.**
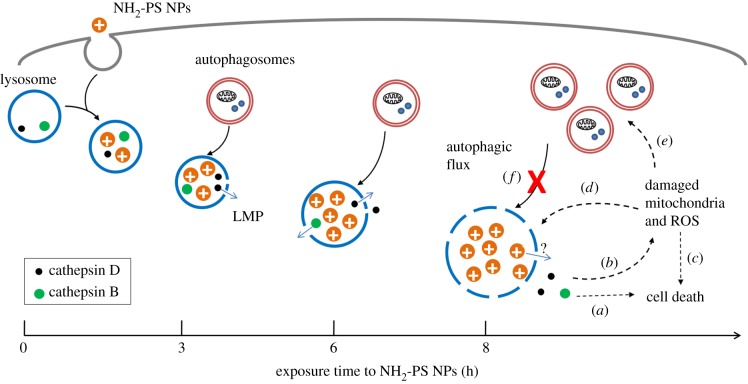
Scheme of LMP and deregulated autophagy induced by NH_2_-PS NPs. NH_2_-PS NPs are endocytosed into lysosomes. After 3 h, these NPs cause mild LMP, leading to leakage of small lysosomal components (processed cathepsin D, 27 kDa) into cytosol. Gradually, after 6 h, the LMP is exacerbated, marked by the release of larger lysosomal components (cathepsin B, 38 kDa). Lysosomal expansion due to the ‘proton sponge' effect of these cationic NPs can be also observed. At this point, the NP-loaded lysosomes might still be functional, and autophagosomes generated from basal level autophagy can still be fused and degraded. However, after 8 h exposure to NPs, lysosomal expansion and LMP continue to aggravate. The released cathepsins can directly lead to caspase-independent cell death (process *a*), or perturb mitochondria (process *b*), resulting into ROS generation and apoptotic cell death (process *c*). The generated ROS can lead to more severe LMP, serving as a feedback loop (process *d*). Autophagy is induced, likely due to the damaged mitochondria and ROS (process *e*). However, the generated autophagosomes cannot be fused with and/or degraded by lysosomes, as they are extensively damaged due to accumulation of NH_2_-PS NPs (process *f*).

We summarize the progression of alternations of lysosomes and autophagy induced by NH_2_-PS NPs in MEF cells in figure 5. NPs accumulate to lysosomes as early as 3 h after exposure and they lead to mild LMP, detected by loss of LysoTracker staining (figure 1*b*,*c*) and release of cleaved cathepsin D (27 kDa), a relatively small component from lysosomes, into the cytosol (figure 2*a*). After 6 h of incubation with NPs, lysosomes have been found to be dramatically expanded, illustrated by increased intensity of LysoTracker (figure 1*a*,*b*,*d*) and enlarged lysosomes (electronic supplementary material, figure S1). At the same time, a larger lysosomal component, cathespin B (38 kDa), can be found in the cytosol, reflecting a larger extent of LMP than that after 3 h exposure time to NPs. At this stage, autophagy is still functional and autophagosomes can fuse with lysosomes for degradation (figures 3*a*,*b* and 4*b*). Eight hours seems to be a critical point after NP treatment, when many signalling events occur. The lysosomal volume is increased even more (figure 1*b*,*d*), accompanied by even more severe LMP (figure 2*a*). The released cathepsins could cleave cytosolic proteins and lead to caspase-independent cell death (figure 5, process *a*), and at the same time they can also perturb mitochondria, resulting into ROS generation that further propagates the cell damage (figure 5, process *b*) [20,36,37,53]. Mitochondrial outer membrane permeabilization is marked as the ‘point-of-no-return' for cell death (figure 5, process *c*). The massive ROS generated by damaged mitochondria could in turn further damage lysosomal membranes, forming a ‘feedback' loop (figure 5, process *d*) [54]. The damaged mitochondria and ROS could induce autophagy to remove the damaged mitochondria (figure 5, process *e*) [55]. However, because the lysosomes are not functional any more, the generated autophagosomes are no longer able to fuse with lysosome and get degraded (figure 5, process *f*), which aggravates NP-induced cell death.

### 3.2. The lysosomal membrane permeabilization induced by NH_2_-PS nanoparticles is progressive

In this study, using two distinct methods, we provide a very refined illustration of LMP induced by NH_2_-PS NPs in MEF cells, where mild LMP can be detected as early as after 3 h exposure to these NPs, probably in a small population of MEF cells that are more sensitive to NPs. The mild LMP can be detected by the loss of LysoTracker dyes already at 3 h exposure to these NPs (figure 1*b*,*c*), preceding cell death, as those cells are still negative to the viability dye PI. It correlates well also with the detection of cleaved cathepsin D (27 kDa), a relatively small component from lysosomes, in the cytosol at 3 h (figure 2*a*). After 6 h exposure, cathepsin B, with higher molecular weight (38 kDa), can be detected in the cytosol as well. This strongly supports that the LMP induced by these NPs is mild and partial at the earlier exposure times to NPs and progressively aggravates with time. This size-selective LMP has been reported earlier, in which smaller FITC-dextran molecules were released from apoptosis-associated LMP, while larger dextran molecules were retained [56]. This early LMP at 3 h is likely to be upstream of all other apoptotic events, as a small percentage of cells are detected as Annexin V+/PI− only at 8 h exposure to NPs.

Our data support that detecting lysosomal content in the cytosol is probably the best way to analyse LMP, as LMP cannot be followed only by flow cytometric analysis of LTG and PI in the later time points (after 6 h). Two hypotheses could be given to explain the disappearance of LTG−/PI− population, which is supposed to represent the cells with LMP, in the later time points. First, we suspect that the global dramatic increase of LTG intensity masks the small decrease of LTG in the cells with mild LMP. Second, these might be the most sensitive cells, which die directly after a few hours of exposure to these NPs. Overall, caution needs to be taken when interpreting flow cytometry results using LysoTracker dyes, because an increase in its intensity (as an example) could be due to an increase in lysosomal volume, lysosomal numbers or their acidity. Other techniques are needed to be able to discriminate these different options.

Finally, NH_2_-PS NPs can cause LMP through three different mechanisms. First, the positive charge of these NPs could damage lysosomal membrane, because they accumulate inside lysosomes and positive charges are known to damage biological membranes [57,58]. Second, lysosomal enlargement induced by these NPs can sensitize lysosomes to LMP, as larger lysosomes are proposed to be more susceptible to rupture [59]. Third, ROS could be an important factor in LMP as lysosomes are highly sensitive to ROS [35]. Increase of ROS has been observed after 3 h exposure to NPs, the same time when NPs have been found accumulating to lysosomes. After 8 h exposure, there is a dramatic increase of ROS, which we think might be related to the extensive LMP, leading to release of lysosomal proteases (cathepsin B and D) which damage mitochondria. Damaged mitochondria could lead to release of ROS, amplifying the ROS level and cell death signalling as discussed above. Our previous data suggest that damage to mitochondria is a downstream process after LMP induced by these NPs. In 1321N1 cells, lysosomal protease inhibitors (pepstatin A and E64d) could retard not only cell death, but also decrease the levels of ROS and caspase 3/7 [14].

We have also observed that the ROS-induced cell death could be partially inhibited by iron chelator DFO. The involvement of iron in the cell death induced by these NPs could be related to ferroptosis, a recently discovered form of regulated cell death [60]. Ferroptosis is characterized by lipid peroxidation dependent on ROS generation and iron availability [61], the involvement of which in the toxicity induced by cationic NPs still needs to be explored.

Lysosomal enlargement after treatment of NH_2_-PS NPs could be due to the ‘proton sponge effect' of these protonatable NPs [16,18]. However, we and others have observed that positive NPs that are unprotonatable could also result in lysosomal swelling [9,18], suggesting that other damages to lysosomal membrane structures (proton pumps, chloride proton exchangers, etc.) could lead to increase of lysosomal volume [19]. Another possible explanation for lysosomal swelling is based on the recent findings on the activation of transcription factor EB (TFEB), a master regulator of lysosomal biogenesis, upon exposure to NH_2_-PS NPs in Hela and PC12 cells [62]. TFEB is probably upregulated as a feedback mechanism to compensate the dysfunctional lysosomes, as was observed in lysosomal storage diseases [63]. However, this possibility remains to be studied in MEF cells after NP treatment.

#### 3.2. Autophagy is induced at the upstream but suppressed at the downstream

Autophagy is a multistep process, including the formation of autophagosomes, fusion between autophagosomes and lysosomes, and degradation of autophagosomes by lysosomes [28]. Our data strongly suggest that NH_2_-PS NPs induce upstream autophagosome formation. However, because lysosomes suffer strong damage due to accumulation of NH_2_-PS NPs, the downstream steps of autophagy, namely fusion between autophagosomes and lysosomes and/or degradation of autophagosomes, are compromised, leading to an overall decrease of the autophagic flux.

Our results show that mTOR-dependent autophagy is activated after 8 h, reflected by the inhibition of pATK at this time (electronic supplementary material, figure S11C). This activation of autophagy might be due to various factors related to the observed lysosomal alterations, as a surviving mechanism to help the cell to adapt from the NP-induced stress (figure 4*c*). First, ROS could directly activate autophagy [55,64]. We have observed a dramatic increase of ROS after 8 h incubation with NPs, the same time when we have observed the inhibition of mTOR and activation of autophagy. Second, we speculate that the released cathepsins could cause damage in mitochondria, which could cue autophagy to remove them through mitophagy, a type of selective autophagy [65]. Third, lysophagy, another type of selective autophagy, could be activated in order to remove the damaged lysosomes [66]. Lastly, the activation of autophagy might be related to the possible upregulation of TFEB as mentioned above. Activation of TFEB could induce lysosomal expansion as well as activation of autophagy [67].

Despite the activation of autophagy as a cell surviving mechanism, the permeabilized lysosomes are no longer functional at the later time points. Therefore, the generated autophagosomes cannot be degraded by lysosome, leading to blockage of autophagic flux. Similar results showing induction of autophagy and reduced autophagic flux have been obtained in some other cell lines after exposure to NH_2_-PS NPs [37,52], and in L-02 and HepG2 cells after treatment of 100 nm silica NPs [68].

It is worth mentioning again that analysing autophagy in a time course allows us to monitor not only the activation/blockage of autophagy at difference stages, but also the rate of autophagic flux over time. At the same time, the time profile allows us to correlate the progression of lysosomal damage with that of autophagy activities. Furthermore, it is strongly argued nowadays that a combination of techniques (such as TEM, fluorescence imaging of autophagy reporters, western blot of markers for autophagy and PI3 K/ATK/mTOR pathway, as were used in this study) needs to be used to in order to follow the highly dynamic process of autophagy [69].

## 4. Conclusion

We demonstrated that NH_2_-PS NPs accumulate to lysosomes in MEF cells, leading to the expansion of lysosomal volume and LMP in a progressive manner, accompanied by release of cathepsins into cytosol and generation of ROS. In a small fraction of cells, mild LMP appears at earlier times without evident lysosome expansion. Autophagy is activated as a surviving mechanism to deal with the NP-induced stress. However, because lysosomes are extensively damaged upon NP treatment, the clearance of generated autophagosomes via lysosomes is greatly compromised and the autophagic flux is decreased. Our data provide new evidence and detailed mechanisms of lysosome and autophagy alternations induced by cationic NPs. Furthermore, our data suggest that it is of great importance to examine the impact of NPs on lysosomes in the context of nanotoxicity, because most NPs accumulate in this compartment, and once there, digestion of their protein corona has been observed. This is also important for drug delivery when lysosome-related pathways (such as autophagy) are the designed target for NPs, as the impact of NPs on lysosomes could dramatically change the course of lysosomal-dependent pathways.

## 5. Material and methods

### 5.1. Cell culture and nanoparticles

The MEF cells were kindly provided by Noboru Mizushima (Tokyo Dental and Medical University, Tokyo, Japan), and 1321N1 cells were obtained from the European Collection of Cell Cultures (ECACC). Cells were routinely cultured in Dulbecco's Modified Eagle's Medium Glutamax (DMEM) (Life Technologies) supplemented with 1% glutamine, 10% heat-inactivated fetal bovine serum (Life Technologies) and 50 units ml^−1^ of penicillin and 50 µg ml^−1^ of streptomycin in a 37°C incubator with 95% air/5% CO_2_ atmosphere. Cells were grown to 70–80% confluency before treatment. Fifty nanometres of blue fluorescently labelled NH_2_-PS NPs was purchased from Sigma-Aldrich and their characterizations can be found in [9,15]. NH_2_-PS NPs were used at 50 µg ml^−1^ for all studies, as has been optimized previously for studies of cell death [9,14,15,33].

### 5.2. Confocal fluorescence microscopy

To examine the colocalization of NPs and lysosomes, MEF cells were treated with NH_2_-PS NPs for indicated time points, followed by staining of 1 µM LTR (Life Technologies) in DMEM for 30 min at 37°C. Stained cells were washed with PBS and fixed with 4% paraformaldehyde (PFA) at room temperature for 30 min, followed by permeabilization with 0.1% SDS at room temperature for 1 h and image acquisition.

To monitor the cellular location of autophagosomes in respect to lysosomes after NP treatment, MEF cells were transfected either with GFP-LAMP1 (kindly provided by J. Lippincott-Schwartz), and RFP-LC3 plasmids together, or with tf-LC3 (both provided by T. Yoshimori), using Lipofectamine 2000 (Life Technologies) according to manufactures' instruction. The transfected MEF cells were exposure to 50 µg ml^−1^ NH_2_-PS NPs for indicated time points. Cells were subjected to imaging immediately without fixation.

All imaging acquisition was carried out with a Leica TCS SP5 confocal laser scanning microscope equipped with 405 nm, 488 nm and 562 nm lasers to excite NPs, GFP and RFP (or LTR), respectively.

### 5.3. Flow cytometry measurement

After exposure to NPs for indicated time points or to positive control *t*-BuOOH for indicated concentrations and time points, cells were harvested by trypsin and stained with 100 nM LTG (Life Technologies) for 15 min at 37°C and 20 µg ml^−1^ PI (Sigma) for 3 min at room temperature. After staining, the cell fluorescence was immediately measured with a Cyan ADP flow cytometer (Beckman Coulter), using a 488 nm laser to excite both fluorophores, FL1 (520 ± 20 nm) band pass filter to collect LTG fluorescence and FL2 (613 ± 20 nm) band pass filter to collect PI fluorescence. The data analysis was carried out using Summit software (DAKO). Gates were set to discriminate cell debris and cell doublets according to the forward and side scattering. The dot plots of PI versus LTG were compensated with proper controls. A quadrat gate in these dot plots was set according to the 0 h untreated cells and used throughout the analysis. The four populations were coloured accordingly to facilitate visualization. Results are representative of three independent experiments, each performed with two replicates. Alternatively, cells were stained with 100 nM LTR and 1 µM TO-PRO 3 and analysed in the same way. The results are presented in the electronic supplementary material.

### 5.4. Cellular fractionation and western blot

The MEF cells were firstly exposed to NPs for indicated times. Separation of cytosolic fractions (lysosome-free) and membrane fractions (containing lysosomes and mitochondria) of MEF cells after treatment of NH_2_-PS NPs was performed as previously described [37]. Twenty micrograms of protein extract was resolved by 12% SDS-PAGE and transferred onto polyvinylidene difluoride (PVDF) membranes. The membranes were blocked for 1 h in PBS-Tween-20 (0.05% (v/v)) containing 5% non-fat milk and probed with primary antibodies against cathepsin B and D (Abcam, Cambridge, MA, USA), followed by incubation with corresponding horseradish peroxidise conjugated secondary antibodies (Sigma). LAMP-1 (Sigma) was used as a control to show no contamination of lysosomes in cytosolic fractions; glyceraldehyde 3-phosphate dehydrogenase (GAPDH) (Cell Signalling) was an endogenous control to show equally loading.

For the other western blot experiments, cells were treated with NPs for indicated time points and cellular proteins were extracted with home-made lysis buffer (50 mM Tris–HCl pH 6.8, glycerol 10% (v/v), 2% SDS (w/v), 10 mM DTT and 0.005% bromophenol blue) and subjected to western blot as previously described [23]. LC3 antibody was purchased from Sigma. Antibodies against p62, pAKT, pS6 were purchased from Cell Signaling (Danvers, MA, USA), and GAPDH from Abcam. Densitometry was performed using ImageJ software (Nationcal Institute of Health).

### 5.5. Treatment of pharmacological compounds and cell death assay

In figure 2*b*, MEF cells were pre-treated with 50 µM pepstatin A (Pep A) or/and 30 µM E64d (Sigma-Aldrich) for 16 h before exposure to NPs. In figure 2*d*, MEF cells were pre-treated with 1 mM DFO for 1 h before NP treatment. In figure 4*c*, MEF cells were pre-treated with 2 mM 3-MA (Sigma-Aldrich), 5 µM wortmannin (Calbiochem) or 200 nM rapamycin (Sigma-Aldrich) for 1 h before NP treatment. All inhibitors were present when cells were exposure to NPs for 16 h, except that DFO was removed before NP treatment. Cells were stained with 20 µg ml^−1^ PI for 3 min before measurement with flow cytometry; 15 000 cells were recorded in each analysis in an EPICS XL flow cytometer (Beckman Coulter, Barcelona, Spain). The percentage of cell death was determined by the percentage of PI positive cells with flow cytometry. The values are the average of two replicates from three individual experiments.

### 5.6. Transmission electron microscopy

The MEF cells treated with NPs for 8 h or untreated were fixed with 3% glutaraldehide and postfixed with 1% osmium and 1.5% potassium ferricyanid. The samples were dehydrated in an ethanol series and embedded in LX 112 resin (Fisher Scientific). Ultra-thin sections of 60 nm were cut and stained with 2% uranyl acetate and lead citrate. Samples were subjected to imaging with a JEOL transmission electron microscope (80 kV) equipped with TemCam-F416 TVIPS camera.

## Supplementary Material

Supplementary Figures

## Data accessibility

The datasets supporting this article are detailed in the electronic supplementary material.
