# Supplementary material for: Lysosome-dependent cell death and deregulated autophagy induced by amine-modified polystyrene nanoparticles
Source: Open Biol. 2018 Apr 11;8(4):170271. doi: 10.1098/rsob.170271 (PMC5936715; doi:10.1098/rsob.170271)
Supplement: Supplementary Figures [file rsob170271supp1.pptx]

## Slide 1
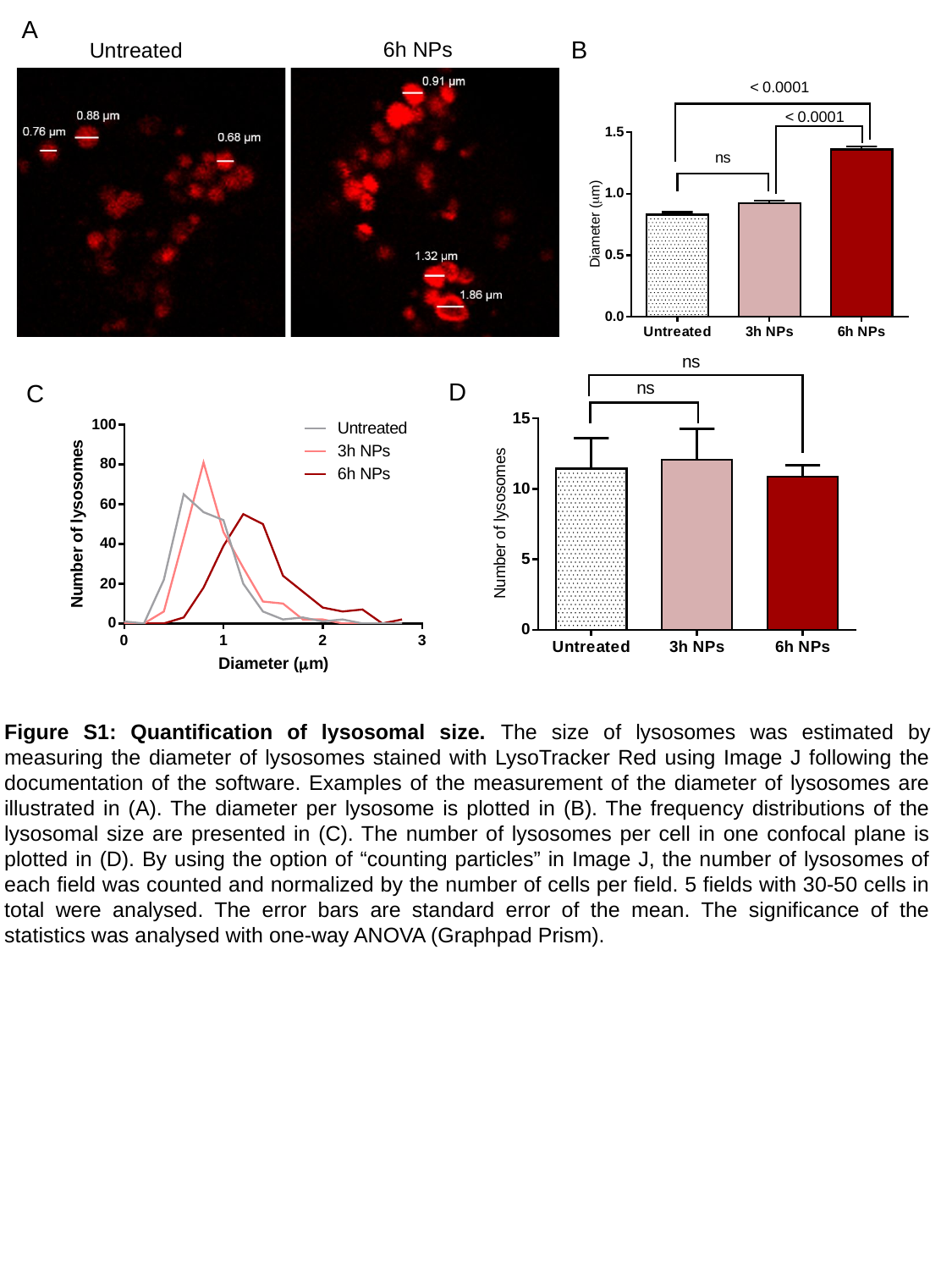

A
B
6h NPs
Untreated
D
C
Figure S1: Quantification of lysosomal size. The size of lysosomes was estimated by measuring the diameter of lysosomes stained with LysoTracker Red using Image J following the documentation of the software. Examples of the measurement of the diameter of lysosomes are illustrated in (A). The diameter per lysosome is plotted in (B). The frequency distributions of the lysosomal size are presented in (C). The number of lysosomes per cell in one confocal plane is plotted in (D). By using the option of “counting particles” in Image J, the number of lysosomes of each field was counted and normalized by the number of cells per field. 5 fields with 30-50 cells in total were analysed. The error bars are standard error of the mean. The significance of the statistics was analysed with one-way ANOVA (Graphpad Prism).

## Slide 2
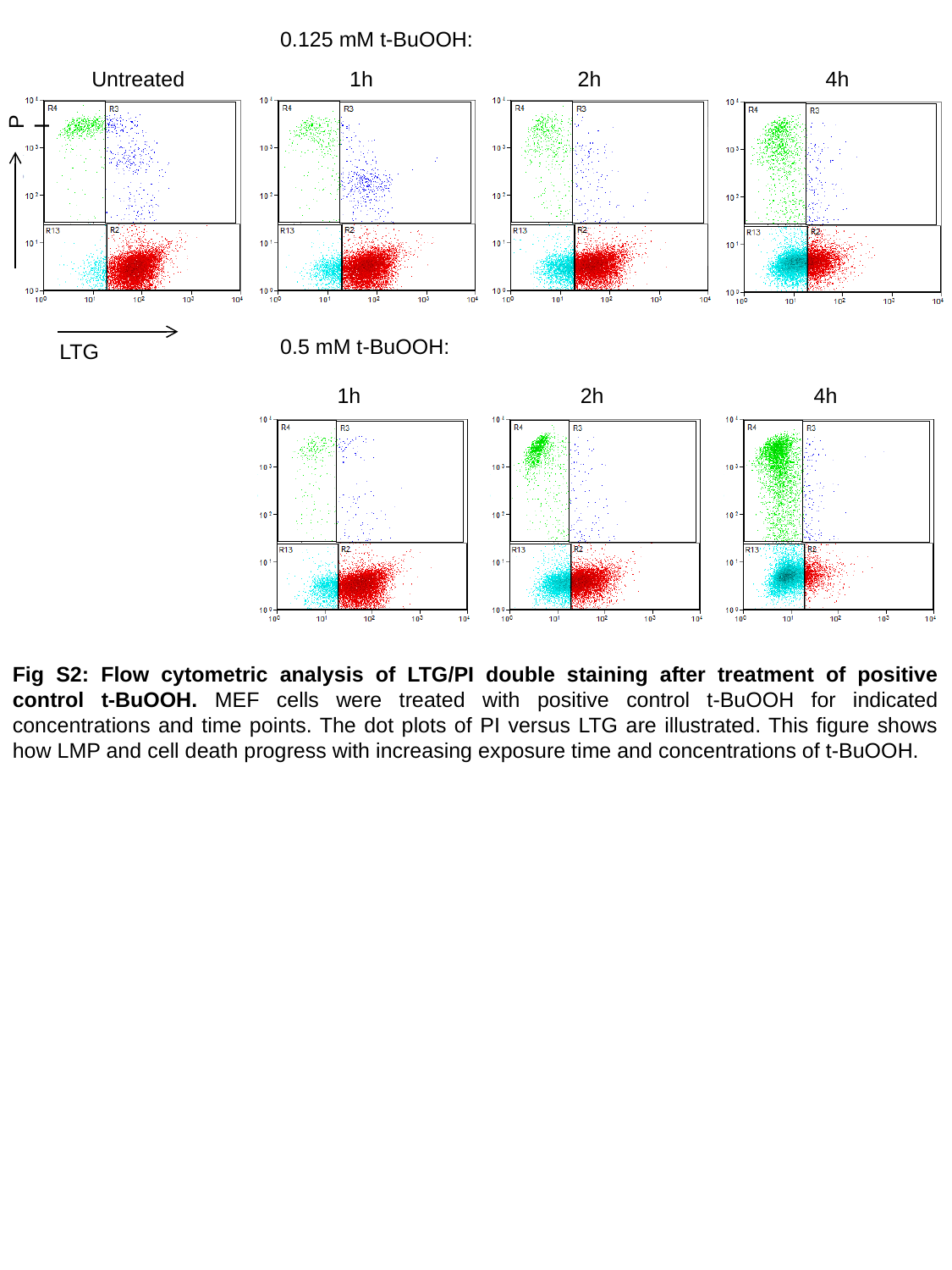

0.125 mM t-BuOOH:
Untreated
1h
2h
4h
PI
LTG
0.5 mM t-BuOOH:
1h
2h
4h
Fig S2: Flow cytometric analysis of LTG/PI double staining after treatment of positive control t-BuOOH. MEF cells were treated with positive control t-BuOOH for indicated concentrations and time points. The dot plots of PI versus LTG are illustrated. This figure shows how LMP and cell death progress with increasing exposure time and concentrations of t-BuOOH.

## Slide 3
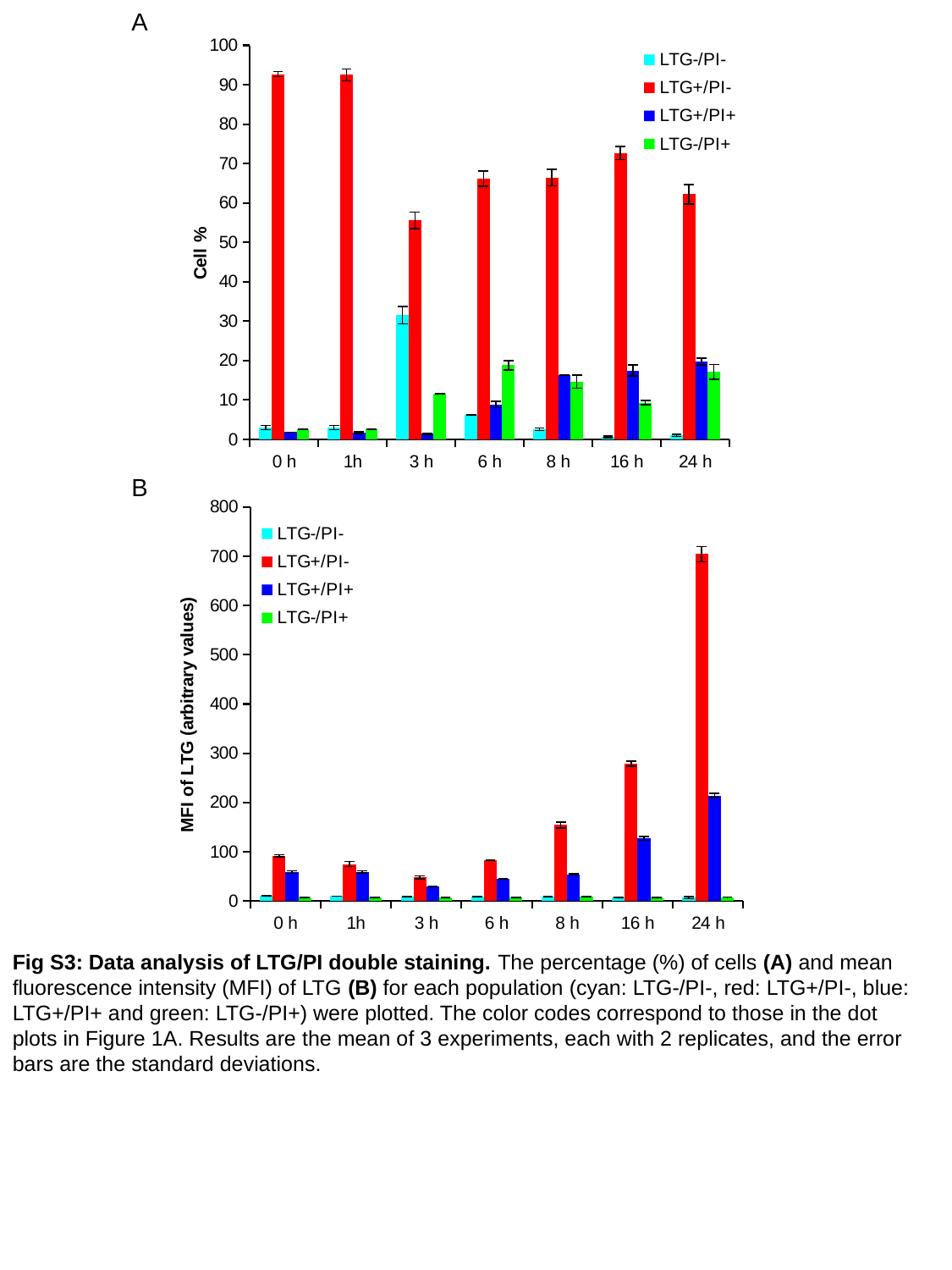

A
### Chart
| Category | LTG-/PI- | LTG+/PI- | LTG+/PI+ | LTG-/PI+ |
|---|---|---|---|---|
| 0 h | 2.965 | 92.65 | 1.8 | 2.585 |
| 1h | 2.9699999999999998 | 92.5 | 1.7 | 2.45 |
| 3 h | 31.485 | 55.565 | 1.405 | 11.545 |
| 6 h | 6.234999999999999 | 66.125 | 8.86 | 18.78 |
| 8 h | 2.555 | 66.43 | 16.28 | 14.635 |
| 16 h | 0.555 | 72.67 | 17.465 | 9.309999999999999 |
| 24 h | 1.0150000000000001 | 62.215 | 19.7 | 17.07 |B
### Chart
| Category | LTG-/PI- | LTG+/PI- | LTG+/PI+ | LTG-/PI+ |
|---|---|---|---|---|
| 0 h | 10.765 | 91.755 | 58.805 | 7.640000000000001 |
| 1h | 9.850000000000001 | 75.0 | 59.0 | 7.75 |
| 3 h | 9.155000000000001 | 47.760000000000005 | 29.65 | 7.305 |
| 6 h | 8.915 | 82.315 | 44.635000000000005 | 7.695 |
| 8 h | 9.455 | 154.16500000000002 | 53.985 | 8.73 |
| 16 h | 7.720000000000001 | 278.54 | 126.83000000000001 | 7.390000000000001 |
| 24 h | 6.815 | 704.335 | 214.18 | 7.82 |Fig S3: Data analysis of LTG/PI double staining. The percentage (%) of cells (A) and mean fluorescence intensity (MFI) of LTG (B) for each population (cyan: LTG-/PI-, red: LTG+/PI-, blue: LTG+/PI+ and green: LTG-/PI+) were plotted. The color codes correspond to those in the dot plots in Figure 1A. Results are the mean of 3 experiments, each with 2 replicates, and the error bars are the standard deviations.

## Slide 4
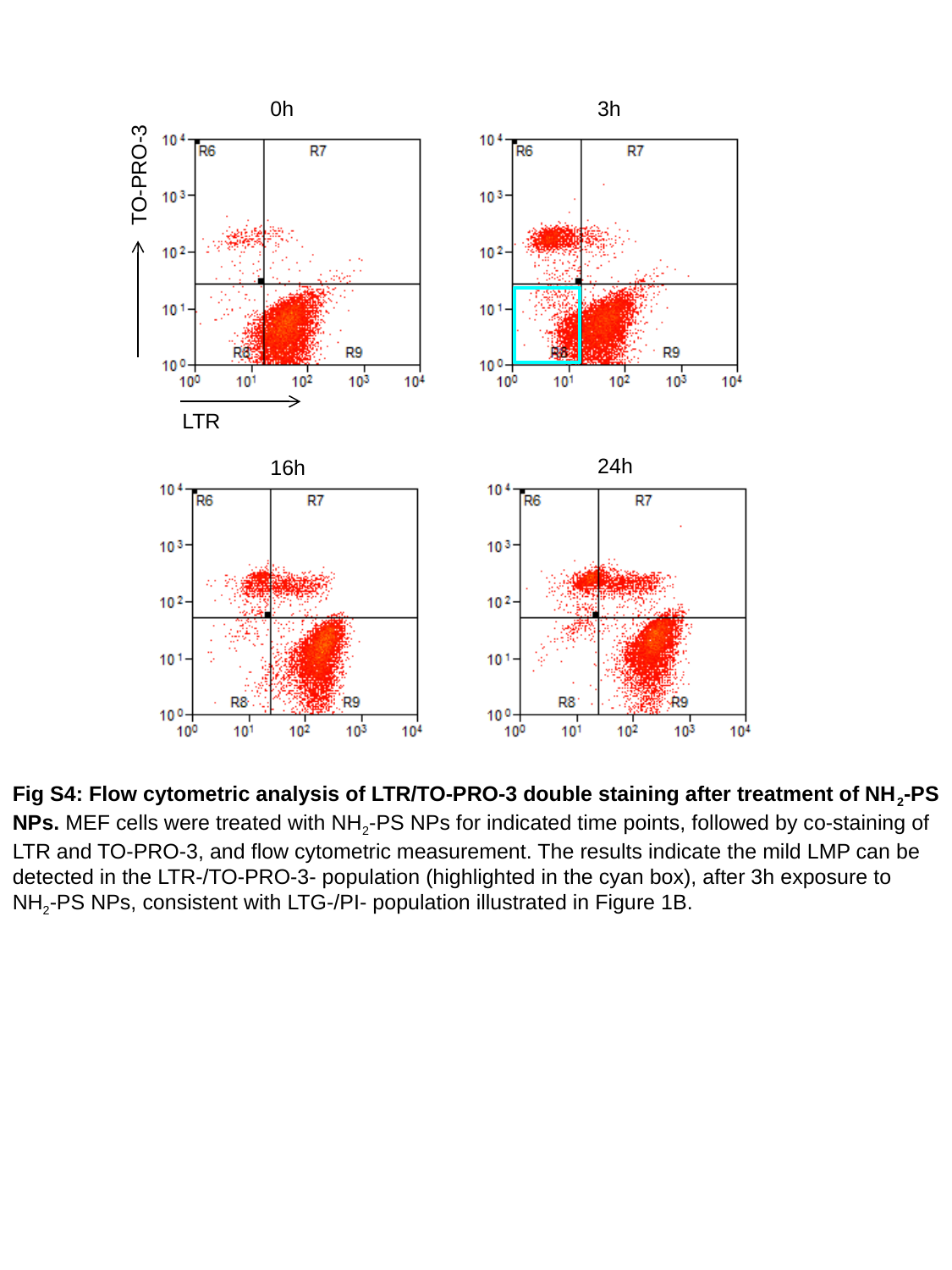

0h
3h
TO-PRO-3
LTR
16h
24h
Fig S4: Flow cytometric analysis of LTR/TO-PRO-3 double staining after treatment of NH2-PS NPs. MEF cells were treated with NH2-PS NPs for indicated time points, followed by co-staining of LTR and TO-PRO-3, and flow cytometric measurement. The results indicate the mild LMP can be detected in the LTR-/TO-PRO-3- population (highlighted in the cyan box), after 3h exposure to NH2-PS NPs, consistent with LTG-/PI- population illustrated in Figure 1B.

## Slide 5
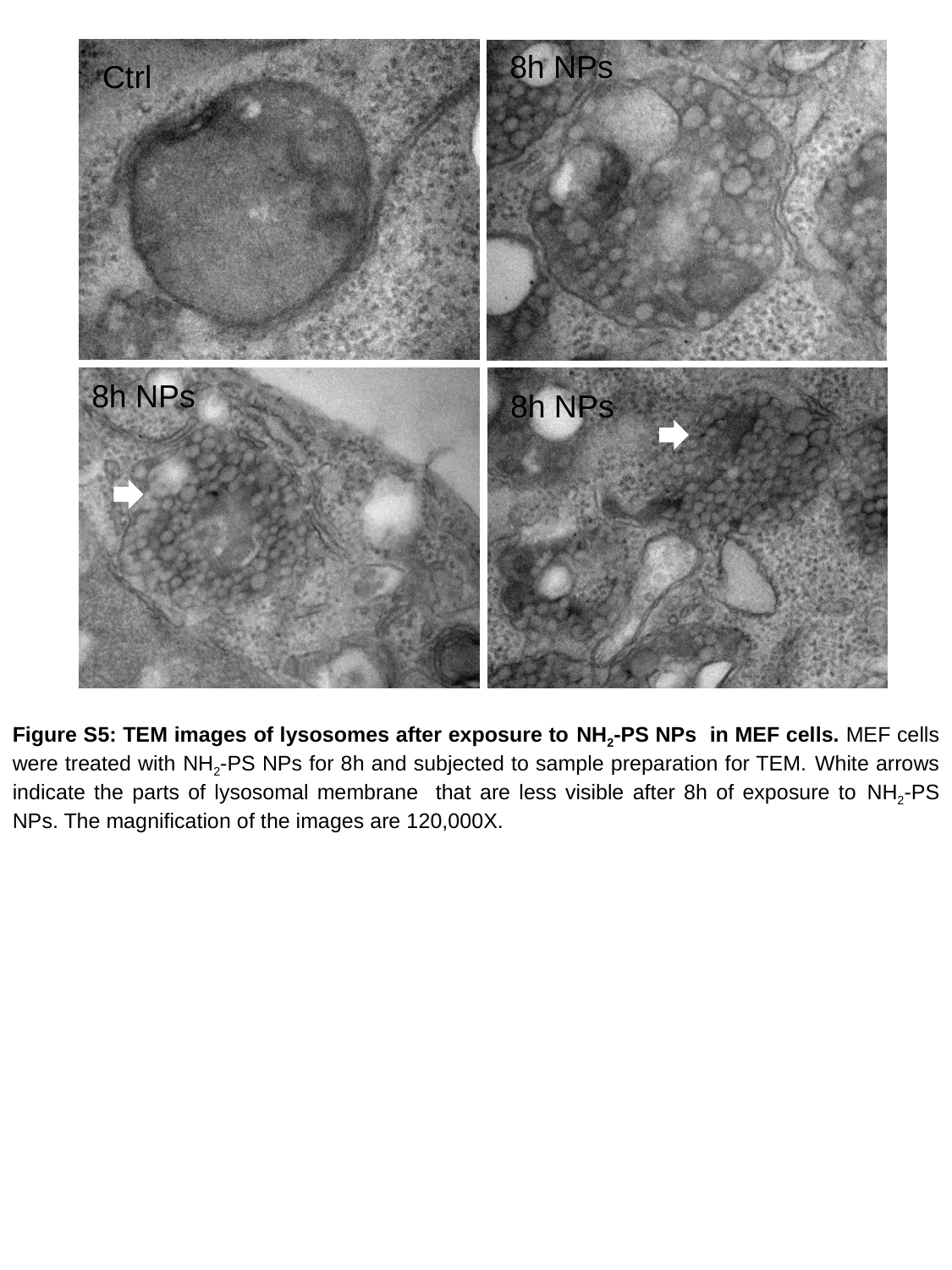

8h NPs
Ctrl
8h NPs
8h NPs
Figure S5: TEM images of lysosomes after exposure to NH2-PS NPs in MEF cells. MEF cells were treated with NH2-PS NPs for 8h and subjected to sample preparation for TEM. White arrows indicate the parts of lysosomal membrane that are less visible after 8h of exposure to NH2-PS NPs. The magnification of the images are 120,000X.

## Slide 6
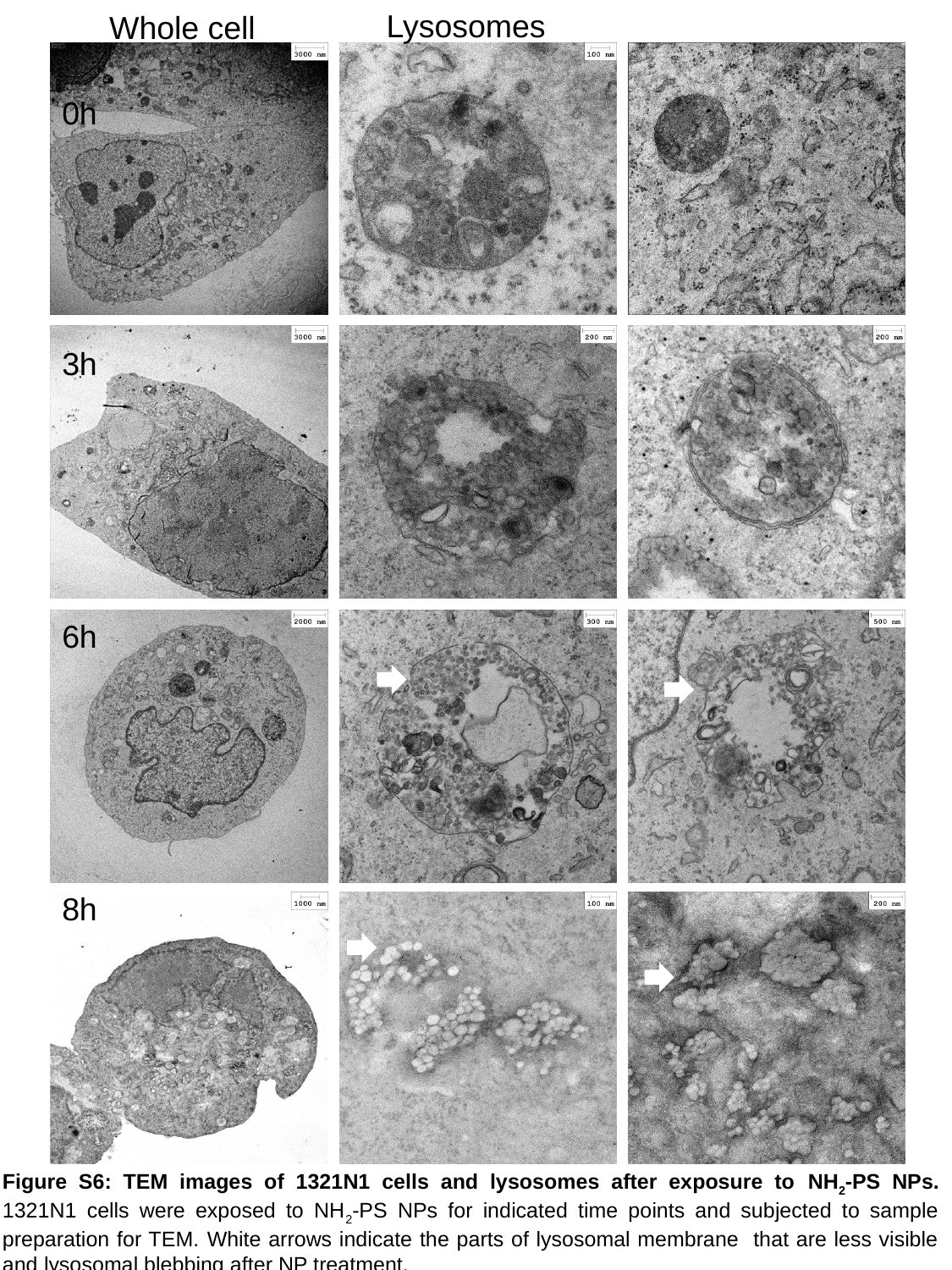

Lysosomes
Whole cell
0h
3h
6h
8h
Figure S6: TEM images of 1321N1 cells and lysosomes after exposure to NH2-PS NPs. 1321N1 cells were exposed to NH2-PS NPs for indicated time points and subjected to sample preparation for TEM. White arrows indicate the parts of lysosomal membrane that are less visible and lysosomal blebbing after NP treatment.

## Slide 7
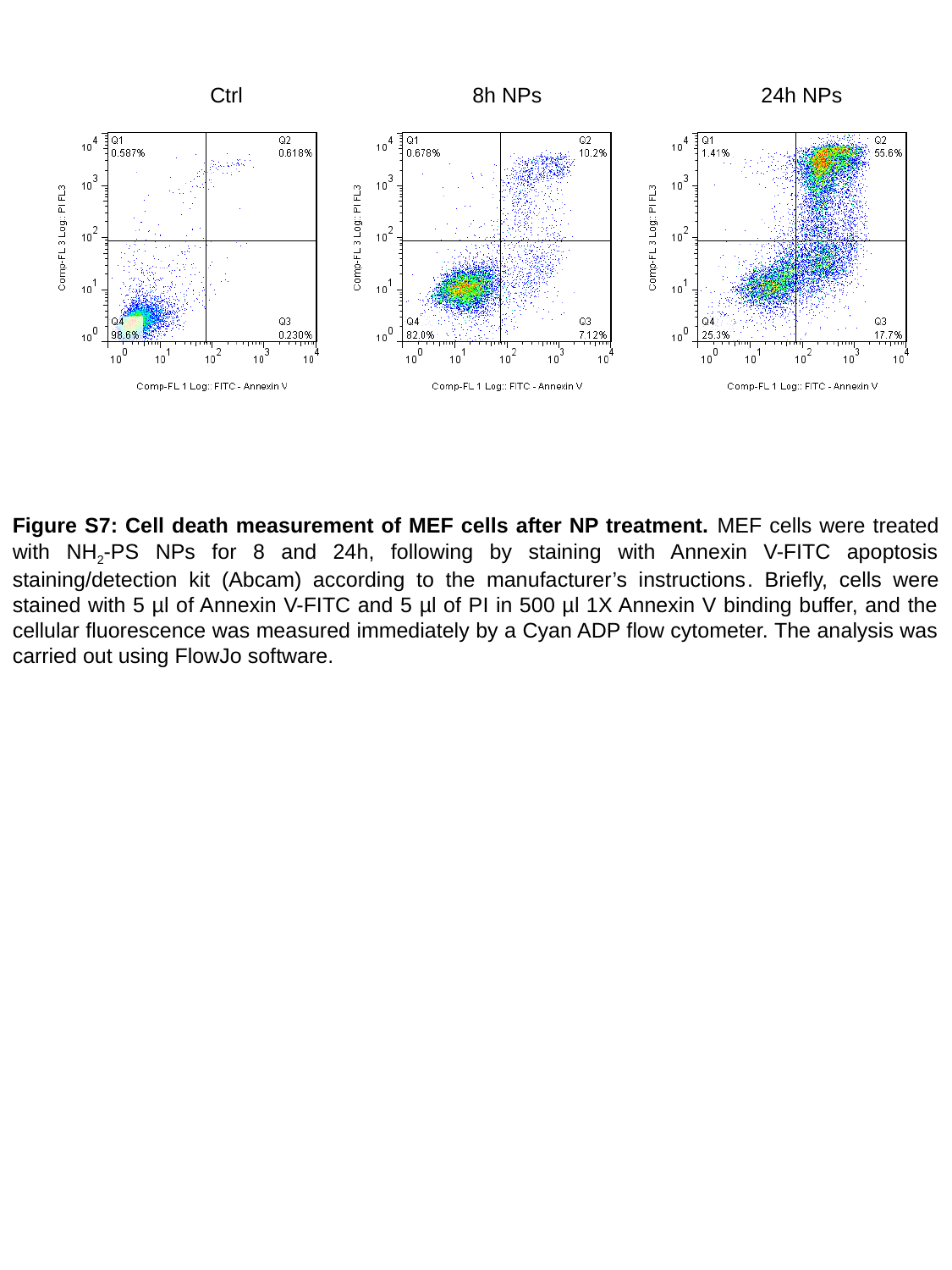

24h NPs
8h NPs
Ctrl
Figure S7: Cell death measurement of MEF cells after NP treatment. MEF cells were treated with NH2-PS NPs for 8 and 24h, following by staining with Annexin V-FITC apoptosis staining/detection kit (Abcam) according to the manufacturer’s instructions. Briefly, cells were stained with 5 µl of Annexin V-FITC and 5 µl of PI in 500 µl 1X Annexin V binding buffer, and the cellular fluorescence was measured immediately by a Cyan ADP flow cytometer. The analysis was carried out using FlowJo software.

## Slide 8
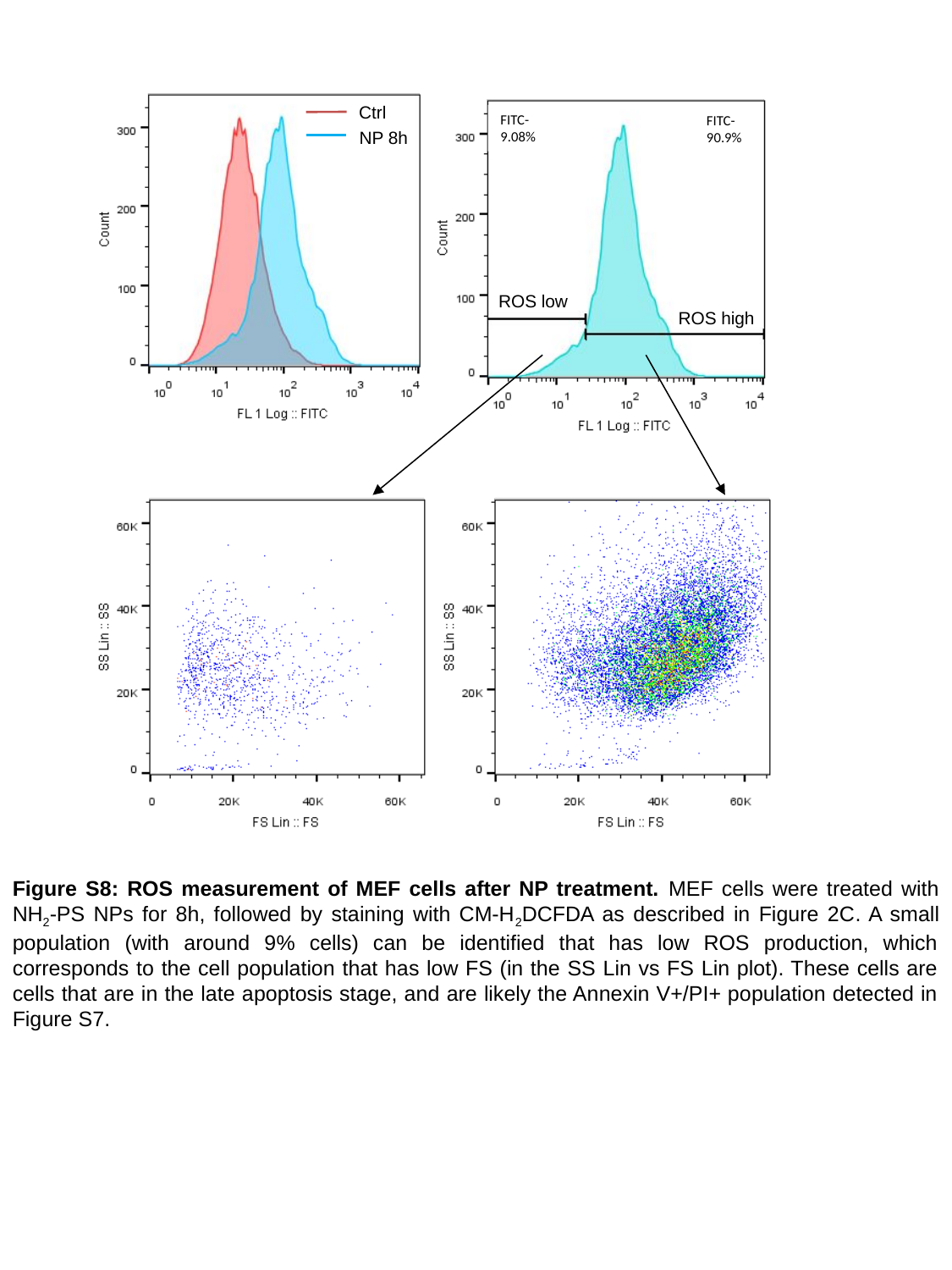

Ctrl
NP 8h
FITC-
9.08%
FITC-
90.9%
ROS low
ROS high
Figure S8: ROS measurement of MEF cells after NP treatment. MEF cells were treated with NH2-PS NPs for 8h, followed by staining with CM-H2DCFDA as described in Figure 2C. A small population (with around 9% cells) can be identified that has low ROS production, which corresponds to the cell population that has low FS (in the SS Lin vs FS Lin plot). These cells are cells that are in the late apoptosis stage, and are likely the Annexin V+/PI+ population detected in Figure S7.

## Slide 9
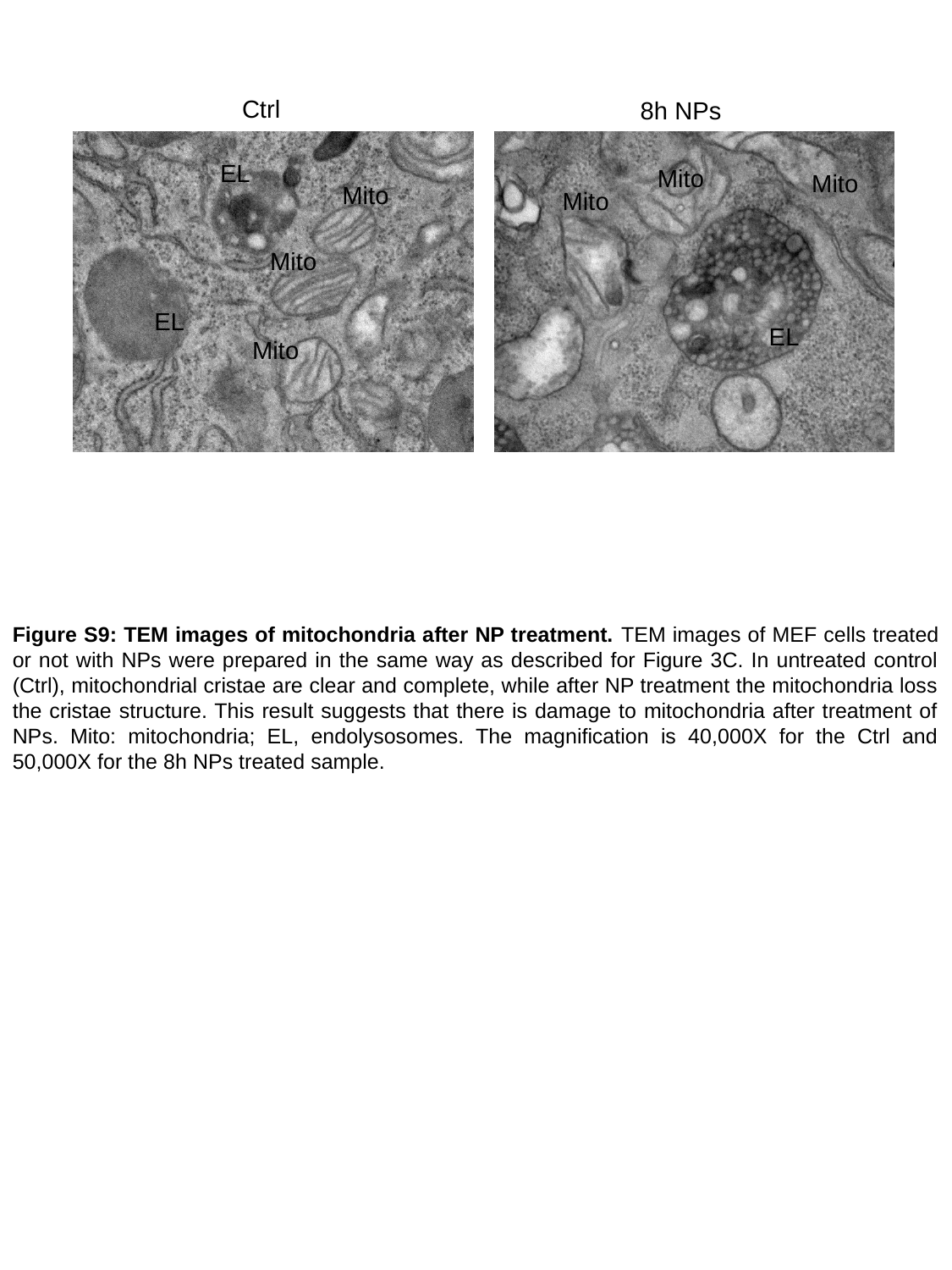

Ctrl
8h NPs
EL
Mito
Mito
Mito
Mito
Mito
EL
EL
Mito
Figure S9: TEM images of mitochondria after NP treatment. TEM images of MEF cells treated or not with NPs were prepared in the same way as described for Figure 3C. In untreated control (Ctrl), mitochondrial cristae are clear and complete, while after NP treatment the mitochondria loss the cristae structure. This result suggests that there is damage to mitochondria after treatment of NPs. Mito: mitochondria; EL, endolysosomes. The magnification is 40,000X for the Ctrl and 50,000X for the 8h NPs treated sample.

## Slide 10
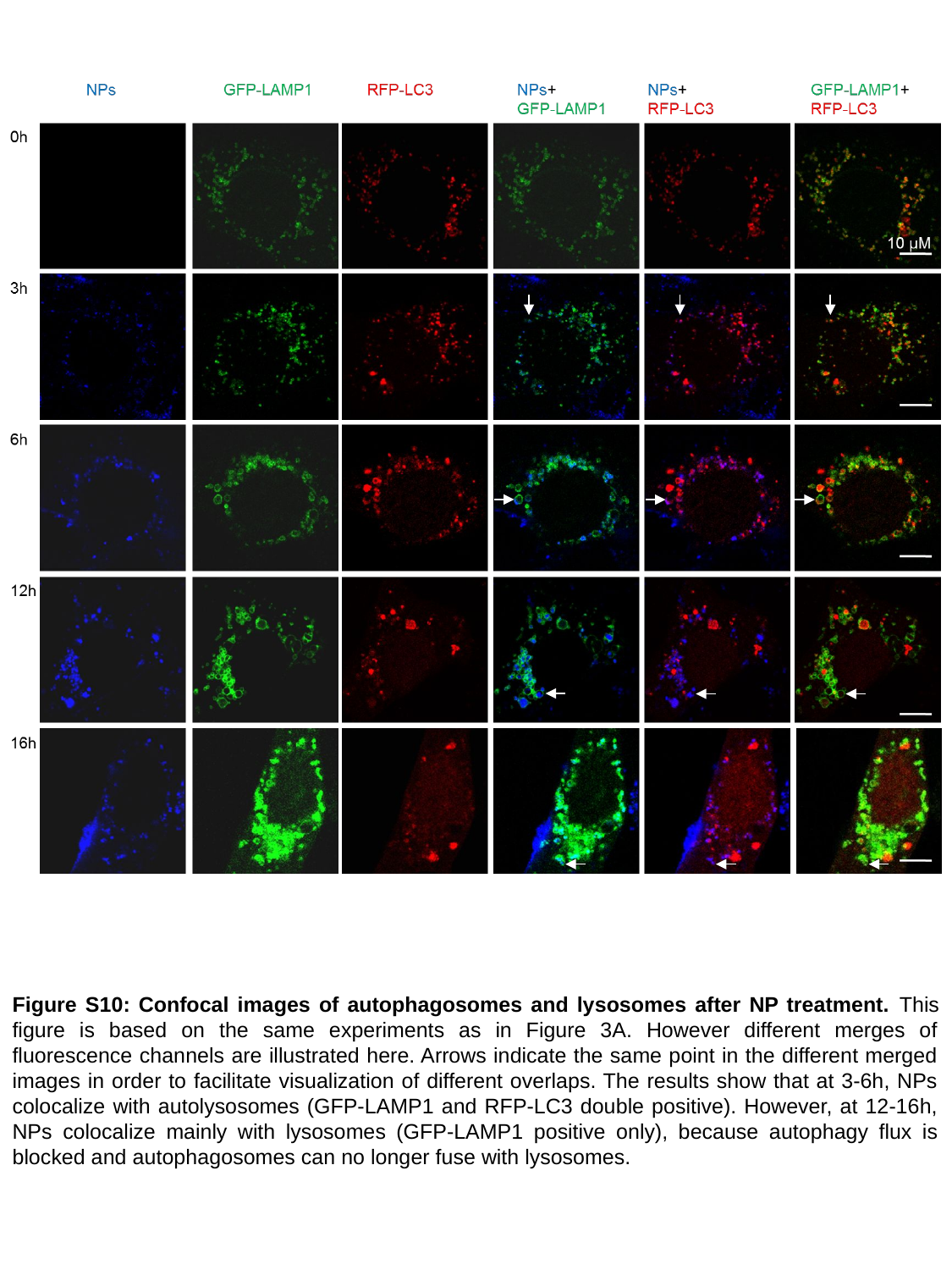

Figure S10: Confocal images of autophagosomes and lysosomes after NP treatment. This figure is based on the same experiments as in Figure 3A. However different merges of fluorescence channels are illustrated here. Arrows indicate the same point in the different merged images in order to facilitate visualization of different overlaps. The results show that at 3-6h, NPs colocalize with autolysosomes (GFP-LAMP1 and RFP-LC3 double positive). However, at 12-16h, NPs colocalize mainly with lysosomes (GFP-LAMP1 positive only), because autophagy flux is blocked and autophagosomes can no longer fuse with lysosomes.

## Slide 11
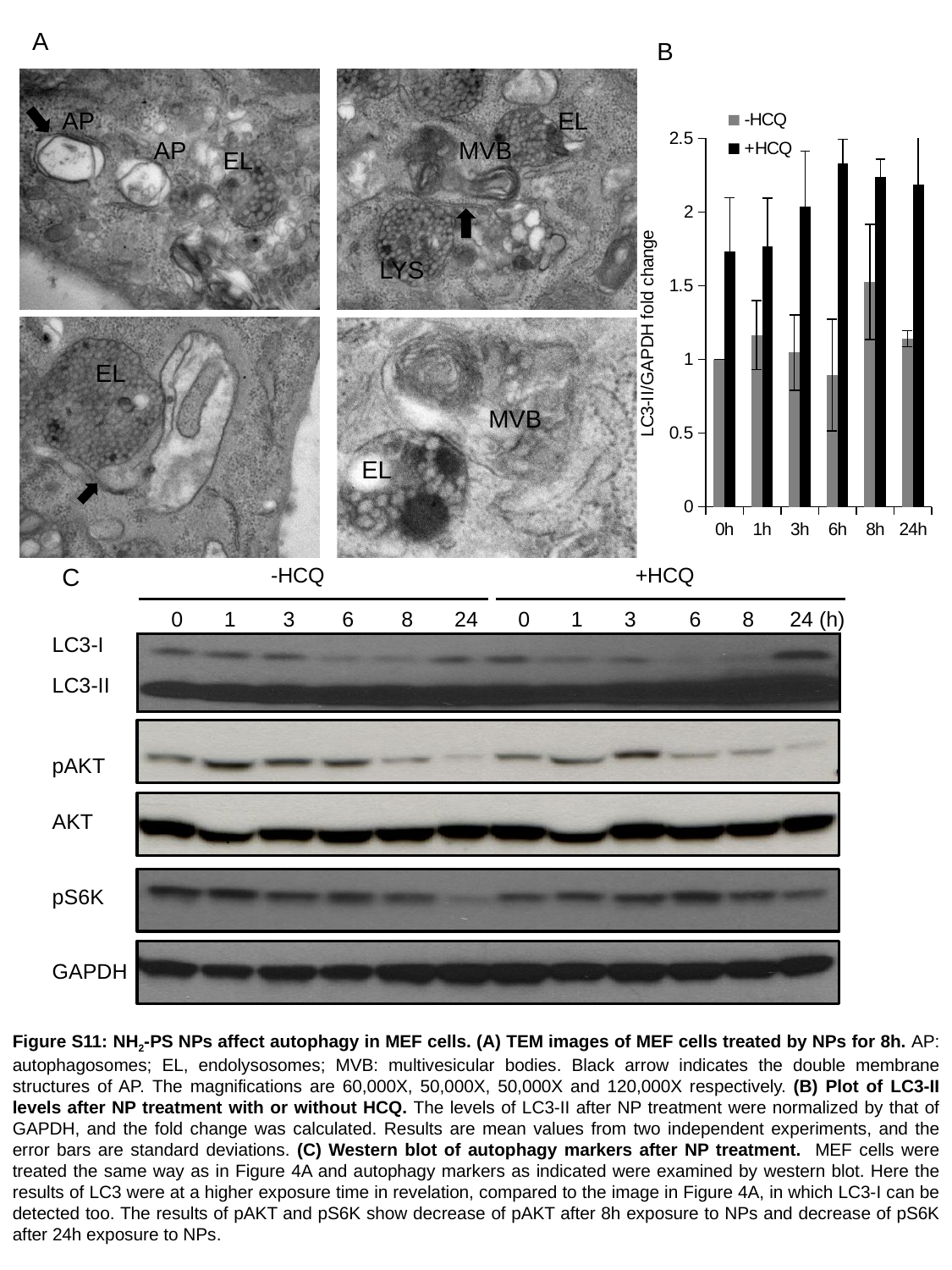

A
B
AP
AP
EL
EL
MVB
LYS
### Chart
| Category | | |
|---|---|---|
| 0h | 1.0 | 1.7303041869680007 |
| 1h | 1.1658015952377103 | 1.7673691165138492 |
| 3h | 1.0464314854241163 | 2.0361130924205715 |
| 6h | 0.8937191074428116 | 2.3303277459040954 |
| 8h | 1.5258330488425598 | 2.2399678794676467 |
| 24h | 1.140799037771136 | 2.1873097299039896 |
EL
MVB
EL
C
-HCQ
+HCQ
0 1 3 6 8 24
0 1 3 6 8 24 (h)
LC3-I
LC3-II
pAKT
AKT
pS6K
GAPDH
Figure S11: NH2-PS NPs affect autophagy in MEF cells. (A) TEM images of MEF cells treated by NPs for 8h. AP: autophagosomes; EL, endolysosomes; MVB: multivesicular bodies. Black arrow indicates the double membrane structures of AP. The magnifications are 60,000X, 50,000X, 50,000X and 120,000X respectively. (B) Plot of LC3-II levels after NP treatment with or without HCQ. The levels of LC3-II after NP treatment were normalized by that of GAPDH, and the fold change was calculated. Results are mean values from two independent experiments, and the error bars are standard deviations. (C) Western blot of autophagy markers after NP treatment. MEF cells were treated the same way as in Figure 4A and autophagy markers as indicated were examined by western blot. Here the results of LC3 were at a higher exposure time in revelation, compared to the image in Figure 4A, in which LC3-I can be detected too. The results of pAKT and pS6K show decrease of pAKT after 8h exposure to NPs and decrease of pS6K after 24h exposure to NPs.

## Slide 12
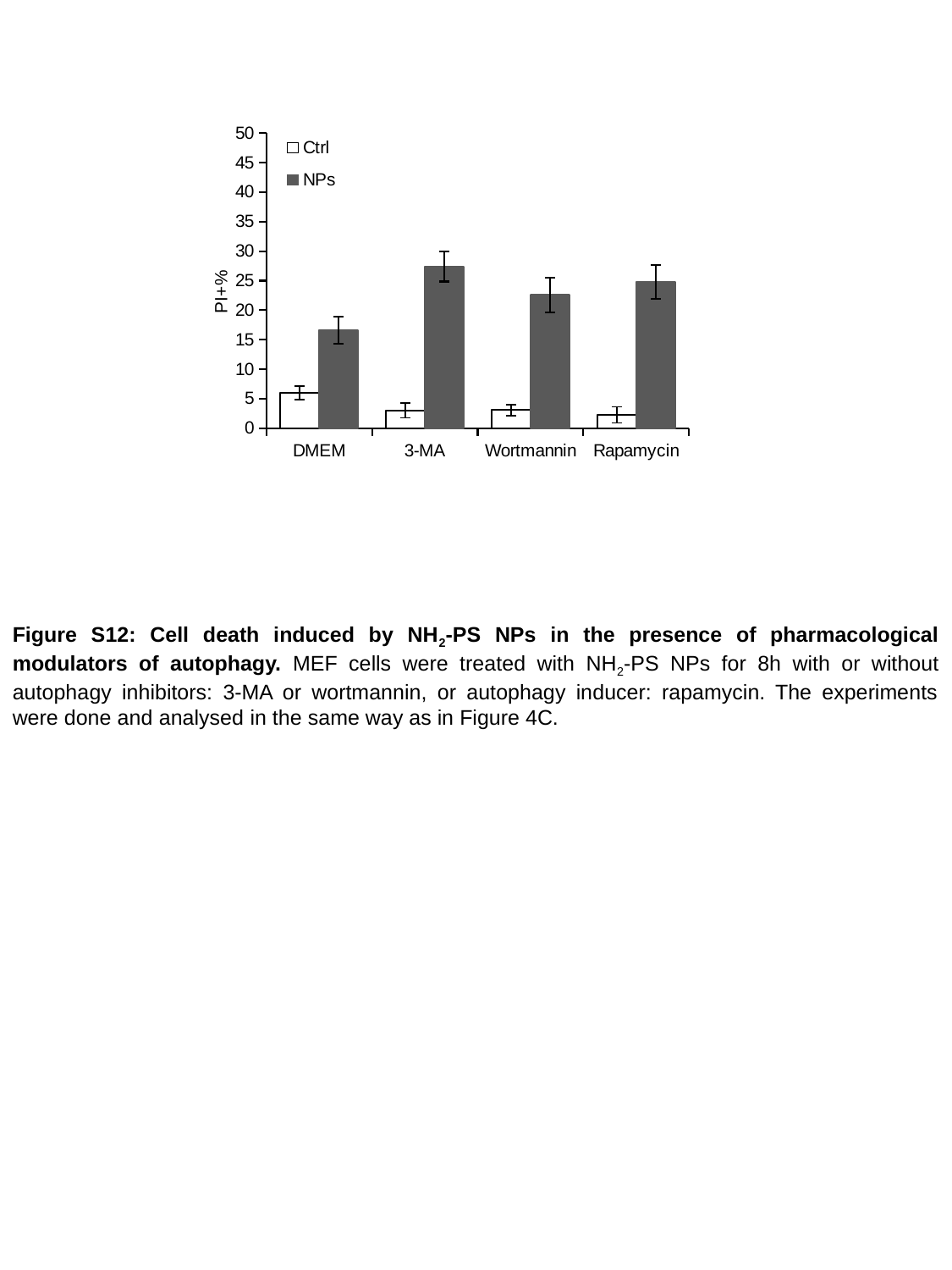

### Chart
| Category | | |
|---|---|---|
| DMEM | 6.0 | 16.6 |
| 3-MA | 3.0 | 27.4 |
| Wortmannin | 3.05 | 22.55 |
| Rapamycin | 2.25 | 24.8 |Figure S12: Cell death induced by NH2-PS NPs in the presence of pharmacological modulators of autophagy. MEF cells were treated with NH2-PS NPs for 8h with or without autophagy inhibitors: 3-MA or wortmannin, or autophagy inducer: rapamycin. The experiments were done and analysed in the same way as in Figure 4C.

## Slide 13
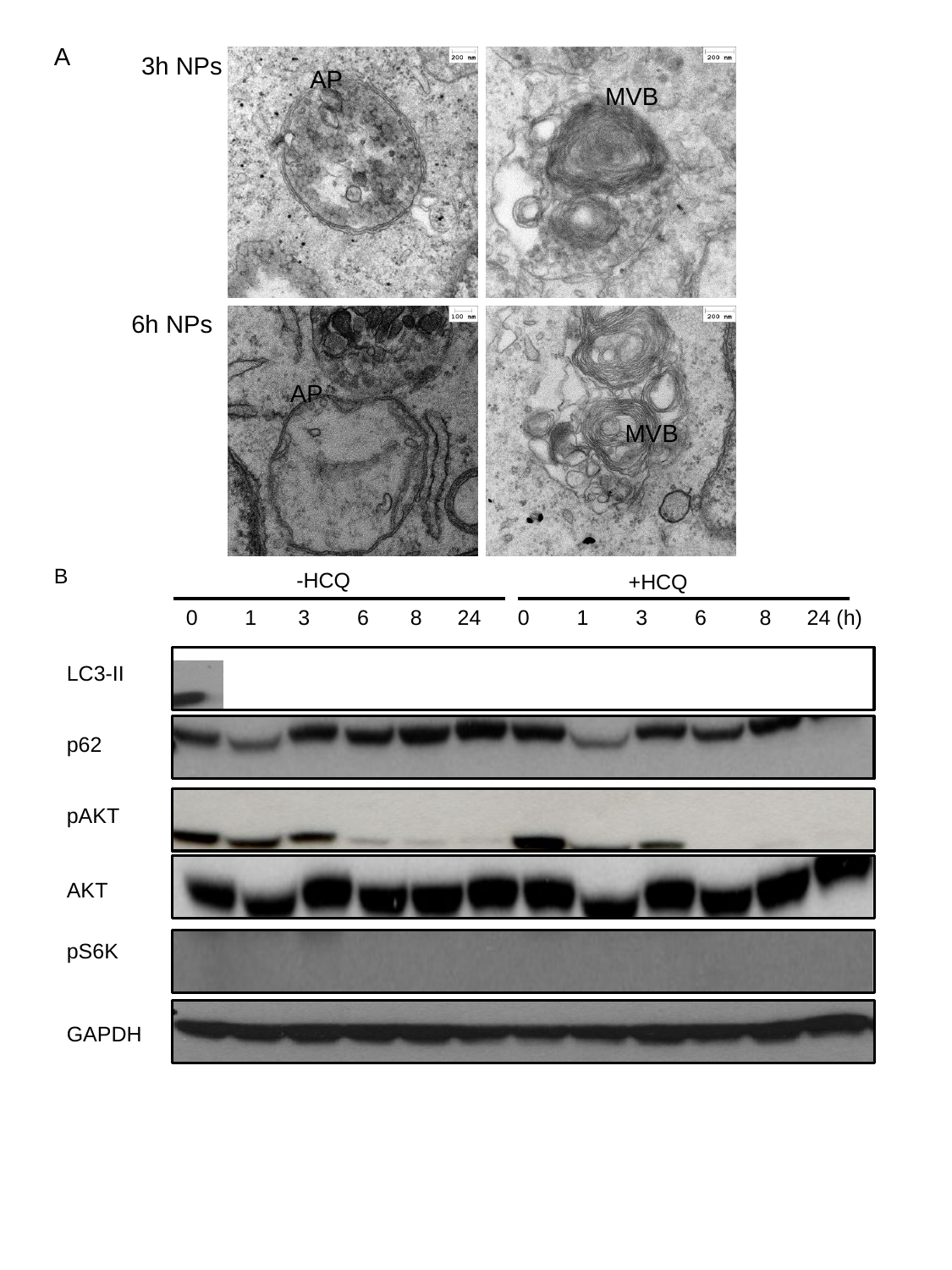

A
3h NPs
AP
MVB
6h NPs
AP
MVB
B
-HCQ
+HCQ
0 1 3 6 8 24
0 1 3 6 8 24 (h)
LC3-II
p62
pAKT
AKT
pS6K
GAPDH

## Slide 14
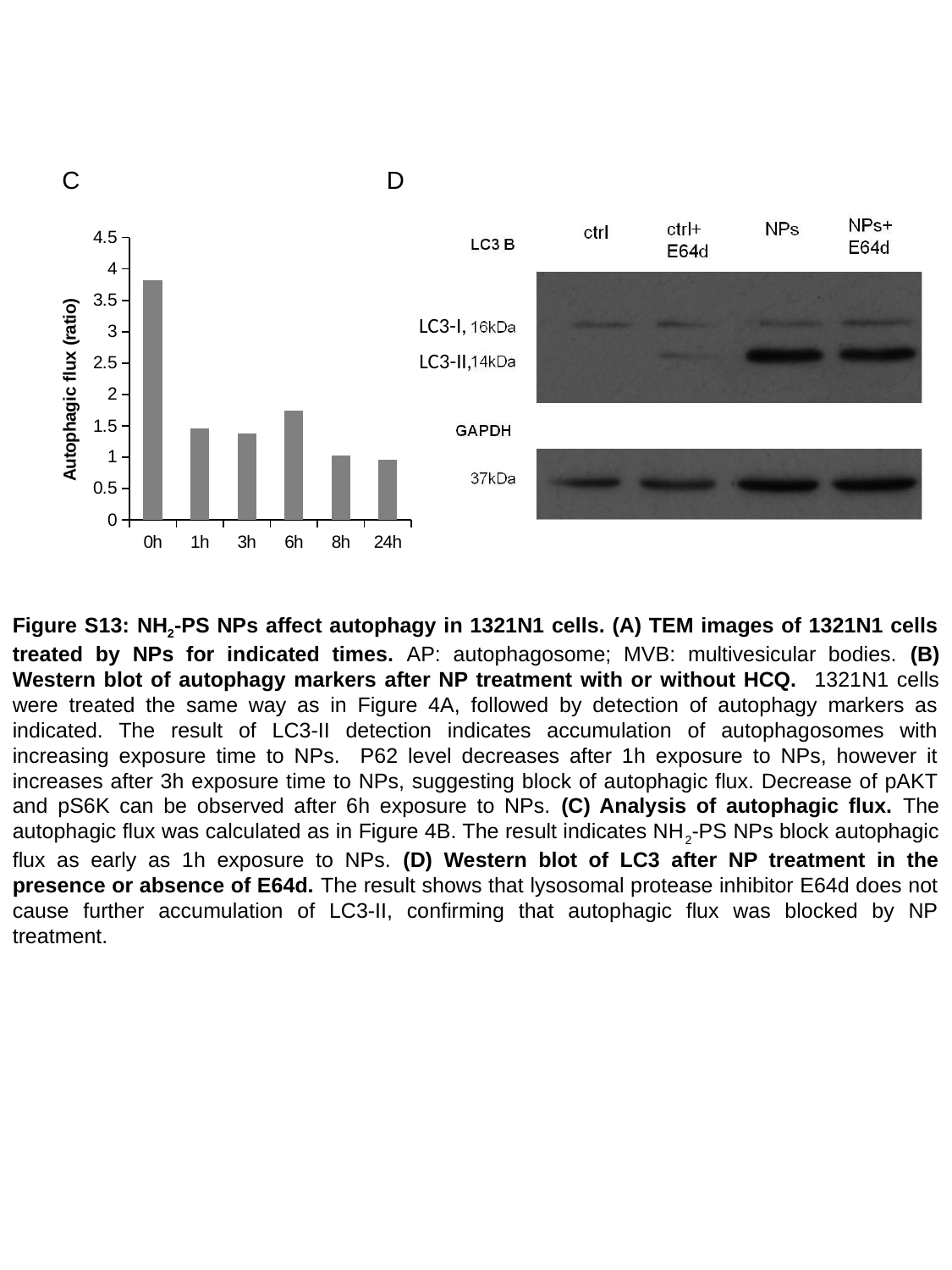

C
D
LC3-I,
LC3-II,
### Chart
| Category | w/o HCQ |
|---|---|
| 0h | 3.8260371235403077 |
| 1h | 1.4514759453003918 |
| 3h | 1.3721701687360541 |
| 6h | 1.7381075237469983 |
| 8h | 1.026296074441606 |
| 24h | 0.9597581386769517 |Figure S13: NH2-PS NPs affect autophagy in 1321N1 cells. (A) TEM images of 1321N1 cells treated by NPs for indicated times. AP: autophagosome; MVB: multivesicular bodies. (B) Western blot of autophagy markers after NP treatment with or without HCQ. 1321N1 cells were treated the same way as in Figure 4A, followed by detection of autophagy markers as indicated. The result of LC3-II detection indicates accumulation of autophagosomes with increasing exposure time to NPs. P62 level decreases after 1h exposure to NPs, however it increases after 3h exposure time to NPs, suggesting block of autophagic flux. Decrease of pAKT and pS6K can be observed after 6h exposure to NPs. (C) Analysis of autophagic flux. The autophagic flux was calculated as in Figure 4B. The result indicates NH2-PS NPs block autophagic flux as early as 1h exposure to NPs. (D) Western blot of LC3 after NP treatment in the presence or absence of E64d. The result shows that lysosomal protease inhibitor E64d does not cause further accumulation of LC3-II, confirming that autophagic flux was blocked by NP treatment.
